# Unveiling the influence of end-capped acceptors modification on photovoltaic properties of non-fullerene fused ring compounds: a DFT/TD-DFT study†

**DOI:** 10.1039/d4ra03170a

**Published:** 2024-06-27

**Authors:** Muhammad Khalid, Noor Fatima, Muhammad Arshad, Muhammad Adeel, Ataualpa A. C. Braga, Tansir Ahamad

**Affiliations:** a Institute of Chemistry, Khwaja Fareed University of Engineering & Information Technology Rahim Yar Khan 64200 Pakistan khalid@iq.usp.br; b Centre for Theoretical and Computational Research, Khwaja Fareed University of Engineering & Information Technology Rahim Yar Khan 64200 Pakistan muhammad.khalid@kfueit.edu.pk; c Industry Solutions, Northern Alberta Institute of Technology Edmonton Alberta Canada; d Institute of Chemical Sciences, Gomal University D. I. Khan Pakistan; e Departamento de Química Fundamental, Instituto de Química, Universidade de São Paulo Av. Prof. Lineu Prestes, 748 São Paulo 05508-000 Brazil; f Department of Chemistry, College of Science, King Saud University Riyadh 11451 Saudi Arabia

## Abstract

Herein, unique A–D–A configuration-based molecules (NBD1–NBD7) were designed from the reference compound (NBR) by utilizing the end-capped acceptor modification approach. Various electron-withdrawing units –F, –Cl, –CN, –NO_2_, –CF_3_, –HSO_3_, and –COOCH_3_, were incorporated into terminals of reference compound to designed NBD1–NBD7, respectively. A theoretical investigation employing the density functional theory (DFT) and time-dependent DFT (TD-DFT) was performed at B3LYP/6-311G(d,p) level. To reveal diverse opto-electronic and photovoltaic properties, the frontier molecular orbitals (FMOs), absorption maxima (*λ*_max_), density of states (DOS), exciton binding energy (*E*_b_), open-circuit voltage (*V*_oc_) and transition density matrix (TDM) analyses were executed at the same functional. Moreover, the global reactivity parameters (GRPs) were calculated using the HOMO–LUMO energy gaps from the FMOs. Significant results were obtained for the designed molecules (NBD1–NBD7) as compared to NBR. They showed lesser energy band gaps (2.024–2.157 eV) as compared to the NBR reference (2.147 eV). The tailored molecules also demonstrated bathochromic shifts in the chloroform (671.087–717.164 nm) and gas phases (623.251–653.404 nm) as compared to NBR compound (674.189 and 626.178 nm, respectively). From the photovoltaic perspectives, they showed promising results (2.024–2.157 V). Furthermore, the existence of intramolecular charge transfer (ICT) in the designed compounds was depicted *via* their DOS and TDM graphical plots. Among all the investigated molecules, NBD4 was disclosed as the excellent candidate for solar cell applications owing to its favorable properties such as the least band gap (2.024 eV), red-shifted *λ*_max_ in the chloroform (717.164 nm) and gas (653.404 nm) phases as well as the minimal *E*_b_ (0.126 eV). This is due to the presence of highly electronegative –NO_2_ unit at the terminal of electron withdrawing acceptor moiety, which leads to increased conjugation and enhanced the intramolecular charge transfer (ICT) rate. The obtained insights suggested that the designed molecules could be considered as promising materials for potential applications in the realm of OSCs.

## Abstract 2

Herein, unique A–D–A configuration-based molecules (NBD1–NBD7) were designed from the reference compound (NBR) by utilizing the end-capped acceptor modification approach.

## Graphical abstract

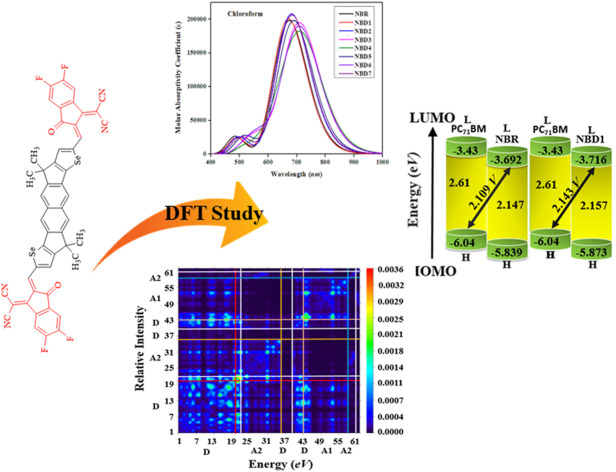

## Introduction

An efficient approach to address the current energy problem is to utilize renewable and environment friendly energy sources such as solar energy to replace polluting fossil fuels.^1^ The OSCs have attracted significant attention over inorganic and conventional silicon-based solar cells, owing to their advantages such as mechanical flexibility, low-cost and light-weight.^2–5^ Some OSCs used fullerene-based acceptors which exhibit considerable advantages with almost 10% power conversion efficiency (PCE).^6^ Despite the remarkable success attained in fullerene-based OSCs, they encountered various challenges, such as expensive purification, inadequate stability and limited absorption in the visible wavelength range.^7,8^ In order to overcome these challenges, significant research efforts have been focused on non-fullerene acceptors, particularly non-fullerene small-molecule acceptors (NF-SMAs).^8^ They offer distinct benefits over fullerene derivatives, such as cost-effectiveness, tunable energy levels and effective absorption of visible light.^9^

Recent reports have demonstrated the NF-SMAs as robust alternatives to the fullerene acceptors, achieving comparable power conversion efficiency (PCE).^10^ OSCs utilizing non-fullerene materials made significant advancements, with achieving PCE of more than 13%.^11–14^ NFAs-based OSCs possess unique properties over the fullerene-based acceptors.^15^ They can be used to accurately modify properties like optical absorption, energy levels and crystallization ability up to 13%.^11–14,16^ The heterojunction formed by donor and acceptor moieties serves as the operative basis for organic solar cells (OSCs).^17^ Hence, PCE of solar cells primarily relies on the characteristics of the acceptor and donor materials.^18^ As the donor materials have undergone significant development, the current emphasis is on enhancing the efficiency of acceptor moieties to achieve highly efficient OSCs.^19^ In recent years, significant development is observed in the fused ring NFAs (FR-NFAs), which feature acceptor–donor–acceptor (A–D–A) architecture integrating a ladder-form fused donor core.^20,21^ Employing this molecular design approach, OSCs harvesting over 18% PCE are developed.^5,22^

Therefore, in the proposed study a non-fullerene small molecule acceptor *i.e.*, NTIC is utilized having A–D–A configuration with a central hexacyclic naphthalene-(cyclopentadithiophene) donor core and terminal acceptor group *i.e.*, 2-(2,3-dihydro-3-oxo-1-*H*-inden-1-ylidene) propanedinitrile (INCN).^23^ In A–D–A type non-fullerene molecules, employing the planar π-extended donor core, such as naphthalene (cyclopentadithiophene), has the potential to enhance photon absorption, leading towards improved short-circuit current density (*J*_sc_), hence making it a promising choice for the proposed research.^24^ Donor core shows a planar structure which is beneficial for the π-electron delocalization and intramolecular charge transport (ICT).^25^ This helps to avoid aggregation in the solid form, making it suitable for efficient OSC devices.^23^

**Scheme 1:**
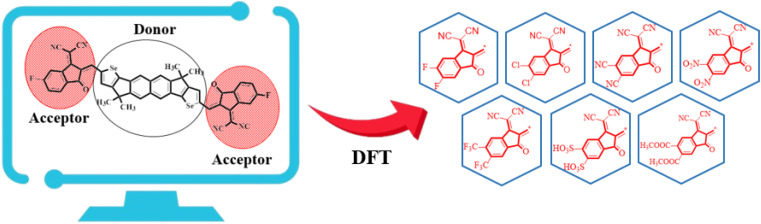
Schematic illustration of the studied compounds (NBR and NBD1–NBD7).

Owing to these facts, NTIC is taken as a parent compound in this research paper. The structural modeling of NTIC into the reference compound (NBR) is accomplished by replacing the sulphur (S) with selenium (Se) atom and 1-methyl-4-hexylbenzene with methyl group to streamline the structure and avoid the computational cost. Consequently, various derivatives, denoted as NBD1–NBD7, are formulated by introducing different acceptor groups at the terminals of the NBR as shown in the Scheme 1. The aim of this research is to examine the influence of various acceptor moieties on the photovoltaic characteristics of the naphthalene-based compound.

The DFT-based calculations are carried out for NBR and NBD1–NBD7 to investigate their FMOs, UV-Vis spectra, DOS, TDM, *E*_b_, *V*_oc_ and fill factor (FF). Literature survey has revealed that the modification of the terminal acceptor moieties used to construct different compounds that shows improved photovoltaic and charge transfer properties. The designed molecules are expected to exhibit remarkable photovoltaic features, including minimum band gap, higher light absorption coefficient and elevated charge mobility.

## Computational procedure

The present theoretical study was conducted utilizing the Gaussian 09 program^26^ and visualization of the outcomes were accomplished *via* the Gauss View 6.0. software.^27^ In order to select a suitable functional for current study, benchmark study was performed between reported experimental and DFT *λ*_max_ values at various functionals. For this purpose, the reference compound (NBR) was first optimized at four different DFT functionals such as B3LYP,^28^ CAM-B3LYP,^28^ MPW1PW91 (ref. 29) and M06 (ref. 30) combining with 6-311G(d,p) basis set. After the successful optimization of NBR, the UV-visible analysis was conducted at the afore-mentioned functionals. The simulated *λ*_max_ values at above-mentioned functionals: B3LYP (674.189 nm), CAM-B3LYP (525.799 nm), MPW1PW91 (637.971 nm) and M06 (635.258 nm) were compared with reported experimental (657 nm)^23^ results to choose an appropriate DFT functional for further investigation. This comparison indicated that B3LYP/6-311G(d,p) level exhibited close harmony with the experimental findings as shown in the Fig. S1† therefore, this functional was selected as the most suitable level for further analyses.

To investigate the photovoltaic and optoelectronic properties of the studied chromophores, their frontier molecular orbitals (FMOs), absorption maxima (*λ*_max_), density of states (DOS), exciton binding energy (*E*_b_), open-circuit voltage (*V*_oc_), transition density matrix (TDM) and fill factor (FF) analyses were performed at B3LYP/6-311G(d,p) level. The global reactivity parameters (GRPs) were calculated using the energies of the FMOs findings (HOMO–LUMO energies). The following software tools were utilized for interpret the data from outputs: PyMOlyze,^31^ Origin 8.0 program,^32^ GaussSum,^33^ Multiwfn 3.7,^34^ Chemcraft^35^ and Avogadro.^36^

## Results and discussion

**Fig. 1:**
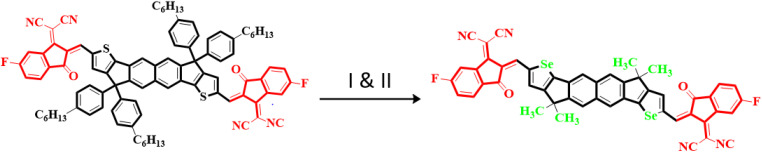
Conversion of NTIC into NBR by (I) replacement of thiophene with selenophene and (II); replacement of hexyl group with methyl group.

In the present study, a donor molecule (NTIC) consisting of A–D–A configuration was employed to develop a reference compound (NBR). The NBR undergoes modification by substituting the sulfur atom (S) with selenium atom (Se) in the fused cyclopentadithiophene ring (donor) of NTIC and replacing its larger bulky alkyl groups (–C_6_H_13_) with smaller methyl unit (–CH_3_) to diminish the steric hindrance and to alleviate computational costs (Fig. 1). The reference chromophores (NBR) consist of two parts: central core (hexacyclic naphthalene-(cyclopentadithiophene)) as donor connected with two terminal acceptor moieties (2-(5,6-flouro-3-oxo-2,3-dihydro-1-*H*-inden-1-y-yildene)propanedinitrile). Various electron withdrawing units –F, –Cl, –CN, –NO_2_, –CF_3_, –HSO_3_, –COOCH_3_ were incorporated into terminals of reference compound to explore the photovoltaic properties. End-capped acceptors play a pivotal role in the design of high-performance OSCs, particularly in donor–acceptor (D–A) conjugated systems. These electron-deficient moieties, attached to the ends of conjugated donor backbones, extend the π-conjugation and facilitate better charge delocalization. This structural modification allows for precise tuning of the HOMO and LUMO energy levels, optimizing the energy gap and enhancing light absorption. In OSCs, end-capped acceptors enable broader absorption spectra, increasing the generation of photogenerated excitons. They also improve exciton dissociation at the donor–acceptor interface, leading to efficient charge separation and transport. Consequently, the incorporation of end-capped acceptors into the molecular architecture significantly boosts the efficiency and durability of organic photovoltaic devices. By the structure tailoring of NBR, seven new derivatives (NBD1–NBD7) were designed with same configuration as that of the parent and reference compounds (A–D–A). The (A–D–A) configuration, representing acceptor–donor–acceptor, is crucial in photovoltaic devices, such as organic solar cells, for several reasons. It enhances light absorption by broadening the absorption spectrum through molecular tuning of acceptor and donor materials. This structure also creates a strong internal electric field that efficiently separates photo generated electron–hole pairs, reducing recombination losses. Additionally, it provides clear pathways for charge carriers, improving charge mobility and minimizing energy losses. These factors collectively boost key solar cell parameters such as power conversion efficiency (PCE), open-circuit voltage (*V*_oc_), short-circuit current density (*J*_sc_), and fill factor (FF), making the A–D–A configuration essential for high-performance solar cells.^27^

**Fig. 2:**
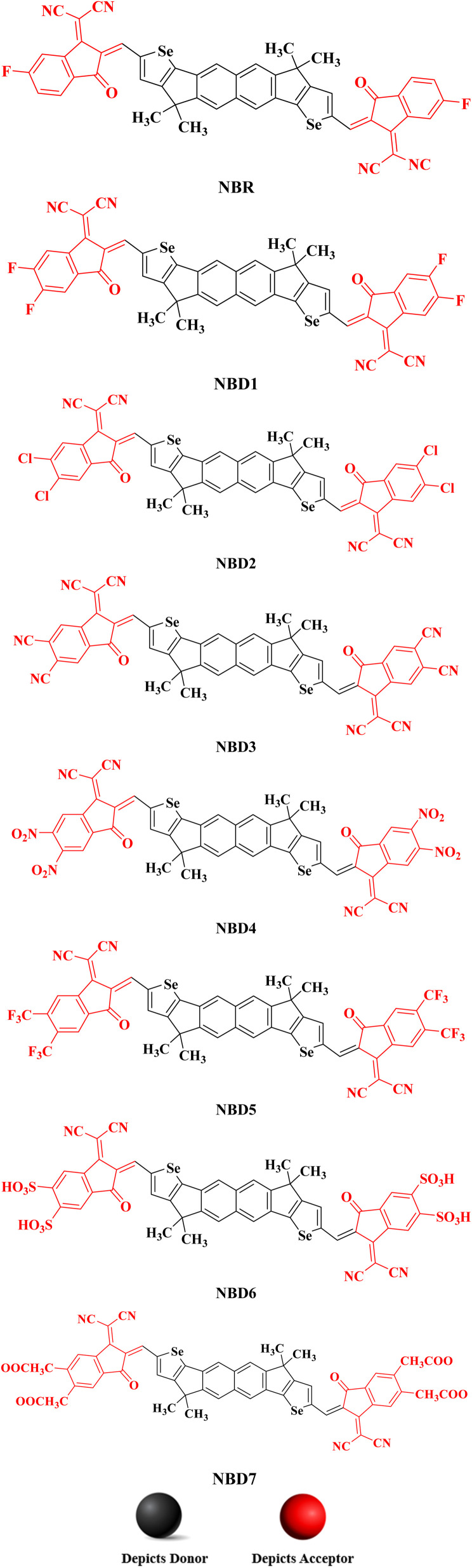
The chemical structures of NBR and NBD1–NBD7 compounds.

All the designed compounds contain hexacyclic naphthalene-(cyclopentadithiophene) core paired with seven distinct acceptor moieties; 2-(5,6-diflouro-3-oxo-2,3-dihydro-1-*H*-inden-1-y-yildene)propanedinitrile (NBD1), 2-(5,6-dichloro-3-oxo-2,3-dihydro-1-*H*-inden-1-y-yildene)propanedinitrile (NBD2), 2-(5,6-dicyano-3-oxo-2,3-dihydro-1-*H*-inden-1-y-yildene)propanedinitrile (NBD3), 2-(5,6-dinitro-3-oxo-2,3-dihydro-1-*H*-inden-1-y-yildene)propanedinitrile (NBD4), 2-(5,6-bis(trifluoromethyl)-3-oxo-2,3-dihydro-1-*H*-inden-1-y-yildene)propanedinitrile (NBD5), 2-(5,6-disulfo-3-oxo-2,3-dihydro-1-*H*-inden-1-y-yildene)propanedinitrile (NBD6) and 2-(5,6- bis(methoxycarbonyl)-3-oxo-2,3-dihydro-1-*H*-inden-1-y-yildene)propanedinitrile (NBD7) attached to it. The Fig. 2 displays their chemical structures, while the optimized geometries obtained *via* the DFT analysis are represented in the Fig. S2.† However, their Cartesian coordinates are provided in the Tables S1–S8 (ESI).†

## Frontier molecular orbitals (FMOs)

**Fig. 3:**
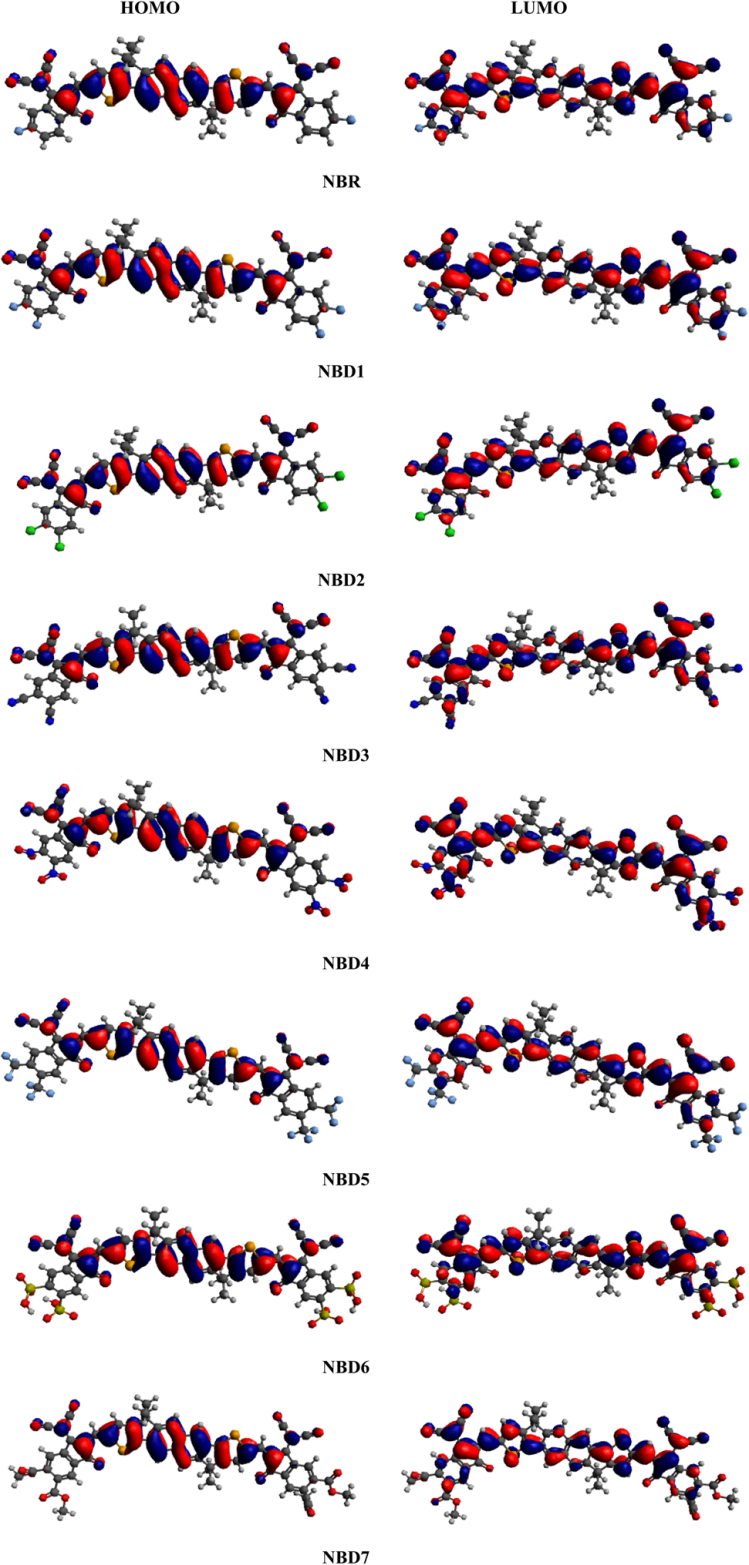
Frontier molecular orbital contour plots for the reference (NBR) and designed molecules (NBD1–NBD7).

Frontier molecular orbitals (FMOs) study is an efficient approach to characterize the photovoltaic properties of the studied molecules. The FMOs diagrams facilitate the understanding of electronic density and the charge distribution pattern of HOMO and LUMO in a molecule.^37^ The HOMO (valence band) serves as an electron donor, while the LUMO (conduction band) acts as an electron acceptor.^38,39^ Effective charge transmission within a molecule requires the transfer of electron density from HOMO to LUMO.^40^ FMOs diagrams of reference (NBR) and tailored compounds (NBD1–NBD7) at B3LYP/6-311G(d,p) level are presented in the Fig. 3. The energy difference between HOMO and LUMO represents the band gap (Δ*E* = *E*_HOMO_ − *E*_LUMO_).^41^ The energy gap (Δ*E*) is crucial in evaluating stability, strength, hardness, softness and chemical reactivity of a molecule.^42^ Moreover, it has a significant impact on the working efficiency of an OSC. The efficiency of OSCs increases with a smaller band gap and conversely decreases with a larger band gap.^43^ The charge carrier mobility in designed molecules (NBD1–NBD7) is enhanced by introducing electron withdrawing acceptor moieties. The energy data of HOMO/LUMO orbitals obtained from the FMOs analysis of derivatives are given in the Table 1. Whereas, the HOMO+1/LUMO−1 and HOMO+2/LUMO−2 values along with their visual representation are recorded in the Table S9 and Fig. S3.†

**Table 1:** Energies of the frontier molecular orbitals of the studied compounds in eV

Compounds	*E*_HOMO_	*E*_LUMO_	Δ*E*
NBR	−5.839	−3.692	2.147
NBD1	−5.873	−3.716	2.157
NBD2	−5.906	−3.776	2.130
NBD3	−6.059	−4.014	2.045
NBD4	−6.077	−4.053	2.024
NBD5	−5.987	−3.887	2.100
NBD6	−6.078	−4.034	2.044
NBD7	−5.908	−3.785	2.123

For NBR, the HOMO/LUMO energy values are obtained as −5.839 eV and −3.692 eV, accordingly, resulting in a band gap of 2.147 eV, slightly larger than that observed in its derivatives except NBD1. This band gap showed harmony with reported experimental value (1.82 eV)^23^ indicating the suitable selection of functional. Calculated HOMO energies of (NBD1–NBD7) are obtained as −5.873, −5.906, −6.059, −6.077, −5.987, −6.078 and −5.908 eV, respectively while LUMO energies of these molecules are −3.716, −3.776, −4.014, −4.053, −3.887, −4.034, and −3.785 eV, correspondingly. Consequently, the band gap Δ*E* values for the studied derivative molecules (NBD1–NBD7) are found to be 2.157, 2.13, 2.045, 2.024, 2.100, 2.044, and 2.123 and 4.21 eV. Literature reveals that the naphthalene based compounds with A–D–A configuration demonstrates the excellent results of band gap as compared to the A–D–A configured indacenodithiophene-based acceptor chromophores ranging from (2.245–2.070 eV), indicating more effective structural modifications.^44^ Among all, NBD4 exhibits the least Δ*E* value as compared to the reference and other designed molecules. This might be owing to the presence of electron withdrawing nitro group (–NO_2_) at the end of acceptor moiety (2-(5,6-dinitro-3-oxo-2,3-dihydro-1-*H*-inden-1-y-yildene)propanedinitrile). Furthermore, this decrease in Δ*E* can also be attributed to the phenomenon of negative inductive effect (−*I*) and prolonged conjugation within molecule. The prolonged conjugation in aromatic rings results in a significant charge transference from donor to acceptor moieties. Additionally, the energies and HOMO/LUMO band gap of NBD4 that showed least band gap among all designed derivatives at B3LYP functional was also investigated at PBE1PBE/6-311 G (d,p) functional. The comparative study between PBE1PBE/6-311 G (d,p) and B3LYP functionals showed that the Δ*E* value of NBD4 determined at PBE1PBE functional is 2.292 eV, higher than the Δ*E* value of 2.024 eV obtained at B3LYP functional.

Moreover, Δ*E* value of NBD1 is reported as 2.157 and this might be due to the incorporation of fluorine (–F) atoms at the terminal acceptors (2-(5,6-diflouro-3-oxo-2,3-dihydro-1-*H*-inden-1-y-yildene)propanedinitrile), which showed positive inductive effect (+*I*) and causes steric hindrance. Accordingly, second narrow band gap is seen in the case of NBD3 and NBD6, probably attributed to the existence of efficient electron withdrawing substituents such as: –CN and –SO_3_H group at the terminal end of acceptor units (2-(5,6-dicyano-3-oxo-2,3-dihydro-1-*H*-inden-1-y-yildene)propanedinitrile) and (2-(5,6-disulfo-3-oxo-2,3-dihydro-1-*H*-inden-1-y-yildene)propanedinitrile) respectively. The –CN and –SO_3_H groups attracted more electrons towards itself, causes an extended conjugation and increased the charge carrier mobility. Similarly, NBD2, NBD5, and NBD7 also exhibits smaller band gap than NBR, but slightly larger than other derivatives, owing to the lesser resonance effect in the molecule. The decreasing order of Δ*E* value for the reference and all derivatives are: NBD1 > NBR > NBD2 > NBD7 > NBD5 > NBD3 > NBD6 > NBD4. From above discussion, it is found that our designed molecules showing remarkable performance in comparison to the reference molecule.^45^

## Chemical reactivity parameters (CRPs)

The HOMO–LUMO band gap is utilized to compute the global reactivity parameters (GRPs) such as: ionization potential (IP),^46^ chemical potential (*μ*),^47^ electron affinity (EA), electronegativity (*X*),^48^ global electrophilicity index (*ω*),^49^ global hardness (*η*),^50^ global softness (*σ*)^51^ and charge transfer index (Δ*N*_max_).^52^ Koopmans' theorem^53^ is widely used for the calculation of these parameters by employing the eqn (S1)–(S8),† and the outcomes of these parameters are presented in the Table 2.

**Table 2:** Calculated GRPs for the studied compounds (NBR and NBD1–NBD7)

Compounds	IP	EA	*X*	*η*	*μ*	*ω*	*σ*	Δ*N*_max_
NBR	5.839	3.692	4.766	1.074	−4.766	10.57	0.466	4.4376
NBD1	5.873	3.716	4.795	1.079	−4.795	10.66	0.464	4.4439
NBD2	5.906	3.776	4.841	1.065	−4.841	11.01	0.469	4.5455
NBD3	6.059	4.014	5.037	1.023	−5.037	12.41	0.489	4.9237
NBD4	6.078	4.053	5.065	1.012	−5.065	12.66	0.494	5.0049
NBD5	5.987	3.887	4.937	1.050	−4.937	11.61	0.476	4.701
NBD6	6.077	4.034	5.056	1.022	−5.056	12.51	0.489	4.9471
NBD7	5.908	3.785	4.847	1.062	−4.847	11.06	0.471	4.5640

The IP and EA represents the electron donating and electron accepting properties of molecules, respectively.^54^ The higher values of IP (6.078–5.873 eV) and EA (4.053–3.716 eV) of designed derivatives (NBD1–NBD7) as compared to NBR (5.839, 3.692 eV, respectively), showed the greater charge transference between donor and acceptor moieties. The decreasing order for both IP and EA values are: NBD4 > NBD6 > NBD3 > NBD5 > NBD7 > NBD2 > NBD1 > NBR.

There exists a close relationship between the GRPs and energy gaps.^55^ Higher kinetic stability of a molecule correlates with a larger energy band gap between HOMO/LUMO.^56^ Chemical potential, global electrophilicity, softness and hardness play a pivotal role in influencing the reactivity, stability and polarizability rate of molecules. Molecules with a narrow band gap may be regarded as soft, chemically reactive, less stable and *vice versa*. All the designed molecules elucidate greater softness (*σ*) and lower hardness (*η*) values than reference molecule which indicates higher reactivity with high polarizing power. The increasing order of *σ* for the titled compounds is as follows: NBD1 (0.464) < NBR (0.466) < NBD2 (0.469) < NBD7 (0.471) < NBD5 (0.476) < NBD3 (0.489) < NBD6 (0.489) < NBD4 (0.494) in eV^−1^. For *η*, the following decreasing order is observed: NBD1 (1.079) > NBR (1.074) > NBD2 (1.065) > NBD7 (1.062) > NBD5 (1.050) > NBD3 (1.023) > NBD6 (1.022) > NBD4 (1.012) in eV. Moreover, the molecules with more negative of chemical potential (*μ*) and high global electrophilicity (*ω*) value are less stable and more reactive. It is concluded from the above discussion that the compound (NBD4) is anticipated to be the most favorable molecule due to its highest softness value (0.494 eV^−1^) and lowest hardness (1.012 eV), which aligns with its lowest energy gap (2.024 eV). The comparative study of global hardness and softness at above mentioned both functionals showed that NBD4 at PBE1PBE level exhibits a lower global softness value (0.436 eV^−1^) and a higher global hardness value (1.146 eV) compared to the results from the B3LYP functional (softness = 0.494 eV^−1^ and lower hardness 1.012 eV).

## Density of states (DOS)

The examination of DOS provides further insight into the distribution of electron density. It describes distribution pattern around HOMO and LUMO which is affected by the nature of electron-withdrawing acceptor moieties. Each structure is divided into two segments for DOS interpretation: the acceptor and the donor.^57^ The results of DOS analysis are studied by PyMolyze software. Here, the acceptor contributes: 30.0, 29.7, 30.4, 31.5, 31.5, 30.8, 31.5 and 30.5% to HOMO and 61.1, 60.0, 60.5, 65.0, 69.6, 61.7, 65.7 and 61.1% to LUMO for NBR and NBD1–NBD7, respectively. Similarly, the donor contributes 70.0, 70.3, 69.6, 68.5, 68.5, 69.2, 68.5 and 69.5% to HOMO, while 38.9, 40.0, 39.5, 35.0, 30.4, 38.3, 34.3 and 38.9% to LUMO for NBR and NBD1–NBD7, accordingly as shown in the Table S12.†

**Fig. 4:**
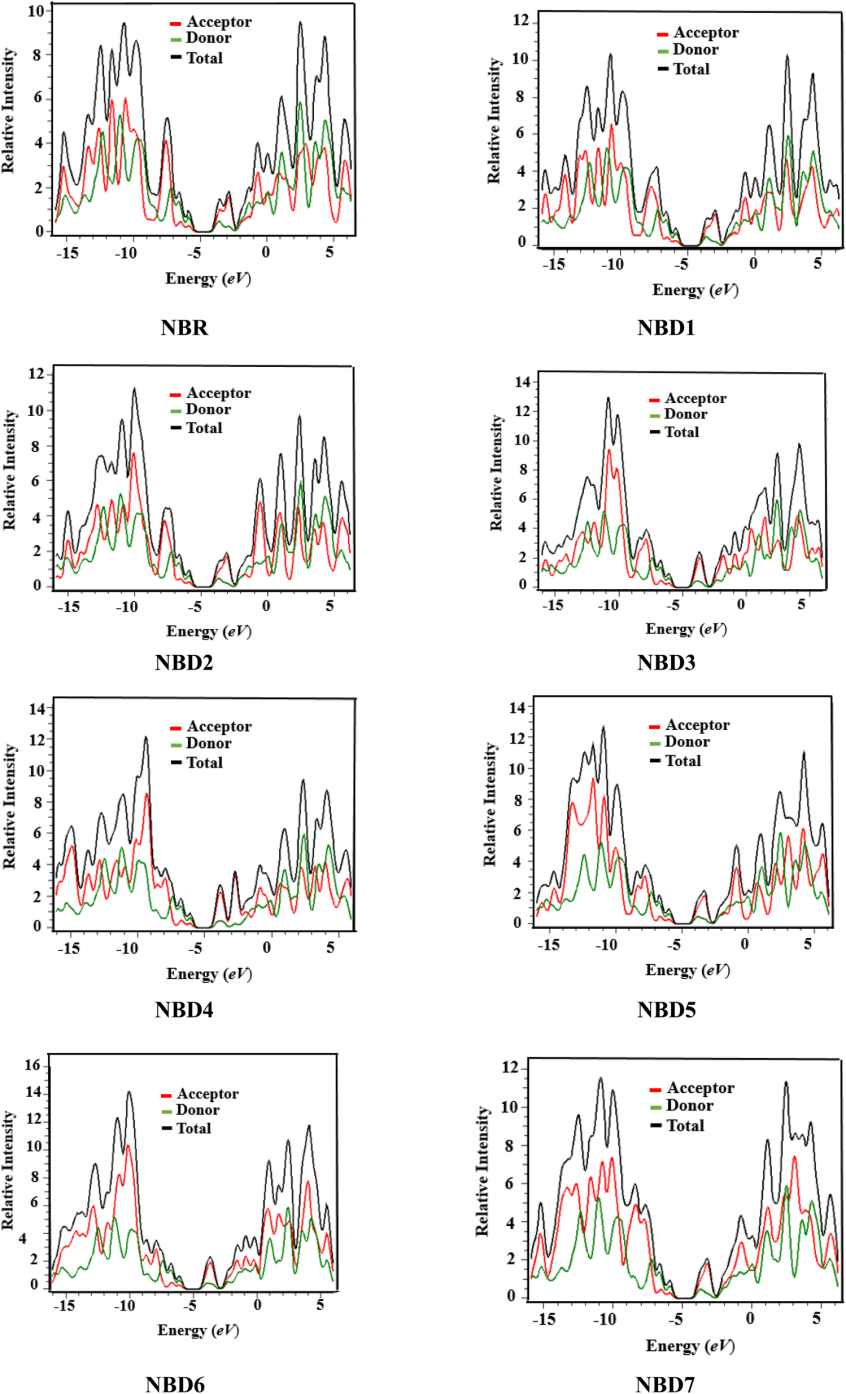
Graphical representation of density of states (DOS) of NBR and NBD1–NBD7.

The DOS graphs represent each part of the molecule in a distinct color *i.e.*, relative intensity of donor is represented in green, while the relative intensity of acceptors is denoted in red color as shown in the Fig. 4. In DOS graphs, the positive values signify LUMO, while, the negative values depict HOMO along the *x*-axis. In the reference molecule (NBR), the HOMO exhibits electron density distributed across the entire molecule, with a slightly higher concentration on the donor groups. Conversely, the LUMO primarily features electron density concentrated on the acceptor moiety, although there is also a portion of electron density associated with the donating groups. In contrast, in the designed compounds (NBD1–NBD7), the HOMO exhibits higher electron density on the cyclopentadithiophene (donor group) and lower density on the acceptor portion. Conversely, the LUMO displays greater electron density on the acceptor group and lesser density on the donor part. These findings demonstrate the end-capped acceptor moieties are satisfactorily proficient in terms of withdrawing effect.

## Absorption properties

**Fig. 5:**
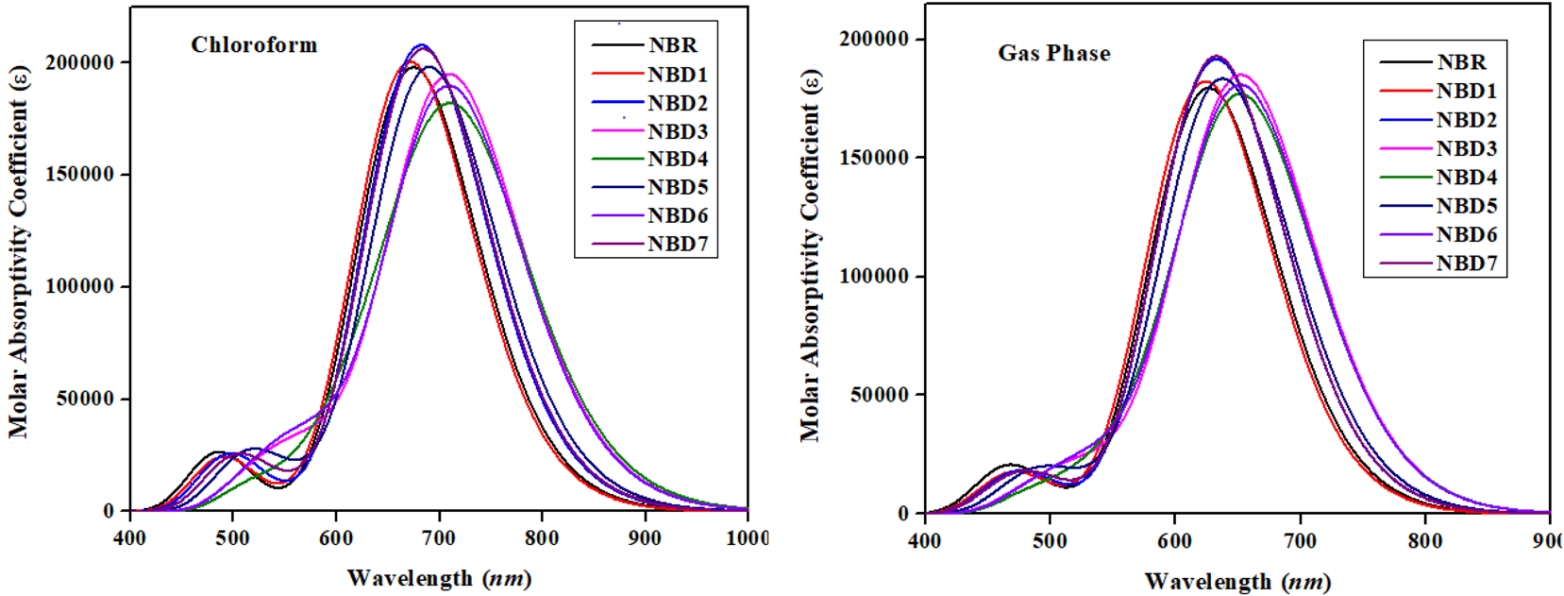
UV-visible spectra of NBR and NBD1–NBD7 in chloroform and gaseous phase.

UV-Vis analysis is used for determining the nature of transitions and charge-transfer characteristics of a molecule.^58^ The photovoltaic properties have correlation with the excitation energy (*E*), oscillation strength (*f*_os_), dipole moment and absorption maxima (*λ*_max_).^59,60^ The value of *λ*_max_ describes the exact energy of photon required for the excitation of an electron from the HOMO towards LUMO, *f*_os_ represents the possibility of transition, while the *E* refers to the energy necessary for a transition to occur.^61^ Therefore, broader absorption at a higher *λ*_max_, high *f*_os_ and low excitation energy are anticipated to yield efficient intramolecular charge transfer (ICT).^62^ The absorption spectra of reference molecule (NBR) and designed compounds (NBD1–NBD7) are calculated in both the gaseous and chloroform solvent phases. The representative values of *λ*_max_ along with their corresponding absorption parameters such as transition energy (*E*), oscillator strength (*f*_os_) and major molecular orbitals contributions are shown in the Table 3. While, the remaining values are recorded in the supplementary part (Tables S10 and S11†). The visual representation of the UV-Vis absorption spectra in both media is shown in the Fig. 5.

**Table 3:** Wavelength (*λ*_max_), excitation energy (*E*), oscillator strength (*f*_os_) and major molecular orbital assessments of the titled compounds (D1–D7) in the chloroform solvent and gaseous phases

	Compounds	DFT *λ*_max_ (nm)	*E* (eV)	*f*_os_	Major MO assessment (%)
aPhase	NBR	674.189	1.839	2.734	H → L (98%)
	NBD1	671.087	1.848	2.768	H → L (98%)
	NBD2	681.827	1.818	2.871	H → L (98%)
	NBD3	709.937	1.746	2.677	H → L (98%)
	NBD4	717.164	1.729	2.357	H → L (98%)
	NBD5	689.562	1.798	2.735	H → L (99%)
	NBD6	710.099	1.746	2.598	H → L (98%)
	NBD7	683.480	1.814	2.848	H → L (98%)
bPhase	NBR	626.178	1.980	2.476	H → L (99%)
	NBD1	623.251	1.989	2.517	H → L (99%)
	NBD2	632.149	1.961	2.646	H → L (99%)
	NBD3	652.132	1.901	2.545	H → L (99%)
	NBD4	653.404	1.898	2.394	H → L (99%)
	NBD5	637.053	1.946	2.531	H → L (99%)
	NBD6	652.372	1.901	2.471	H → L (99%)
	NBD7	632.149	1.961	2.663	H → L (99%)

a Chloroform solvent.

b Gas phase.

All the designed derivatives (NBD1–NBD7) exhibited higher maximum absorption values (*λ*_max_) as compared to the reference (NBR) which might be attributed to the mutual effect of auxochromes and chromophores incorporated in the NBD1–NBD7 compounds. Moreover, the absorption shift towards longer wavelengths is more prominent in case of the chloroform solvent which indicated that polarity induces the enhanced charge generation capacity. The values of *λ*_max_ of NBD1–NBD7 compounds in the solvent chloroform and gas phase are noted in the range of 671.087–717.164 nm and 623.25–653.404 nm, accordingly. Whereas, the *λ*_max_ for reference (NBR) compound in solvent and gas phases are 674.189 and 626.178 nm, respectively. The absorption maxima (*λ*_max_) of NBD1–NBD7 in solvent phase are found in the following decreasing order: NBD4 (717.164) > NBD6 (710.099) > NBD3 (709.937) > NBD5 (689.562) > NBD7 (683.480) > NBD2 (681.827) > NBR (674.189) > NBD1 (671.087) in nm. Similarly, the following absorption maxima trend is seen in gas phase: NBD4 (653.404) > NBD6 (652.372) > NBD3 (652.132) > NBD5 (637.053) > NBD7 (632.149) > NBD2 (632.149) > NBR (626.178) > NBD1 (623.251) in nm. Moreover, the investigated molecules (NBR and NBD1–NBD7) exhibit lower corresponding excitation energies (*E*) as 1.839, 1.848, 1.818, 1.746, 1.729, 1.798, 1.746 and 1.814 eV, respectively, in the chloroform and 1.980, 1.989, 1.961, 1.901, 1.898, 1.946, 1.901 and 1.961 eV in the gas phase, respectively.

Among all the designed molecules, NBD4 exhibits the highest *λ*_max_ in both media (717.164 nm in the chloroform and 653.404 nm in the gas) with lower excitation energy values of 1.729 eV (in chloroform) and 1.898 eV (in gas phase). This might be due to the presence of –NO_2_ group at the terminal acceptor moiety which has strong resonating electron withdrawing effect.^63^ Furthermore, its lower excitation energy leads to an increased charge transference from the HOMO to LUMO. Whereas, NBD1 exhibits the lowest *λ*_max_ of 671.087 nm (in solvent chloroform) and 623.251 nm (in gas phase), with the highest excitation energy (*E*) as 1.848 eV (in solvent) and 1.989 eV (in gas phase). From literature, Asif *et al.* investigated the optical properties of naphtho-dithiophene based non-fullerene acceptor molecule. The results demonstrates that the absorption maximum *λ*_max_ for designed acceptor molecules (NDT1–NDT4) is 430.2 nm, 449.8 nm, 473.9 nm, and 444.9 nm, respectively. While the current research features broader and longer wavelength absorption ranging from (671.087–717.164 nm) demonstrate excellent optical properties. Therefore, it is anticipated that all the designed compounds exhibit significant optical properties, with high efficiency at low excitation energies in the absorption spectrum.

## Transition density matrix (TDM) and exciton binding energy (*E*_b_)

**Fig. 6:**
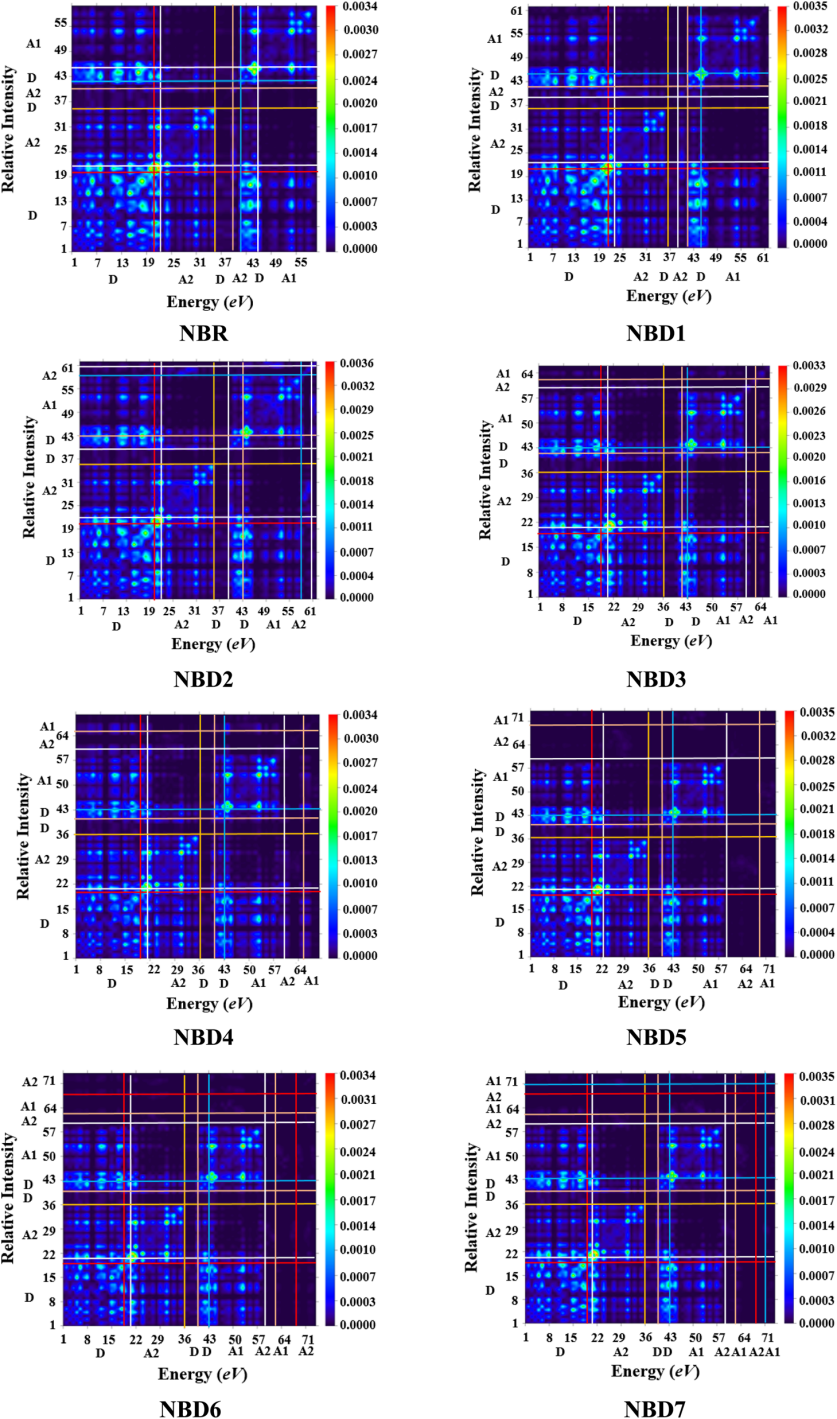
Transition density matrix plots of NBR and NBD1–NBD7 at S_1_ state.

The TDM is employed to estimate and analyze the electronic charge transfer in the excited state. It helps to understand the nature of transitions, hole–electron localization and de-localization as well as the interaction of donor and acceptor units.^64–66^ It offers a comprehensive insights into electronic transitions taking place within a photovoltaic material.^67^ TDM plots for transitions in the first excited state (S_1_) are represented in the Fig. 6.

The hydrogen atoms are mainly neglected by default, due to their minimal contribution to transitions. The investigated molecules are divided into two parts: acceptor (A) and donor (D) whose electron coherence regions are separated by the horizontal and vertical lines. The plots illustrated the accumulation of holes and electrons of the exciton in donor and acceptor part. The electron density in the reference molecule (NBR) is mostly present on the diagonal of the donor part. Whereas, in all the designed compounds (NBD1–NBD7), the distribution of electronic density is mainly concentrated on both the acceptor and donor moieties (mostly on the acceptor part) along diagonal and off-diagonal portions, facilitating the efficient charge transference from donor to acceptor moiety. This efficient charge transfer phenomenon is owed to the prolonged conjugation of utilized acceptor moieties.

Binding energy (*E*_b_) significantly influences the photovoltaic properties, excited separation rate and efficiency of the OSCs.^67^ The *E*_b_ represents the energy needed to dissociate excitons into free charge carriers.^68^ It is performed to calculate the coulombic force of interaction between electrons and holes in a molecule. Also, it is directly proportional to the band gap (*E*_H–L_) and coulombic interaction between electrons and holes and inversely proportional to the exciton dissociation rate.^69^ The lower the binding energy (*E*_b_) and coulombic interactions, the higher will be the dissociation rate in excited state and *vice versa*. The values for *E*_b_ are calculated by using the eqn (1).^70^

1 *E*_b_ = *E*_H–L_ − *E*_opt_

where, *E*_H–L_ demonstrates the HOMO/LUMO energy gap and *E*_opt_ is minimum energy needed for transition from the ground state (S_0_) to excited state (S_1_). The *E*_b_ values for reference and all the designed chromophores are presented in the Table 4. NBD4 exhibits the lowest *E*_b_ (0.126 eV) as compared to NBR reference and all other designed compounds, whereas NBD2 displays the highest binding energy (0.169 eV). Therefore, NBD4 shows the maximum dissociation potential due to weaker coulombic interactions between electron and hole. The decreasing order of binding energy in eV is: NBD2 (0.169) > NBD1 (0.168) > NBR (0.167) > NBD7 (0.162) > NBD5 (0.154) > NBD3 (0.144) > NBD6 (0.143) > NBD4 (0.126). Based on the above discussion, it is inferred that all the designed derivatives (excluding NBD1 and NBD2) demonstrate lesser binding exciton energies and greater charge separation.

**Table 4:** Calculated binding energy (*E*_b_) of titled compounds^a^

Compounds	*E*_H–L_	*E*_opt_	*E*_b_
NBR	2.147	1.980	0.167
NBD1	2.157	1.989	0.168
NBD2	2.13	1.961	0.169
NBD3	2.045	1.901	0.144
NBD4	2.024	1.898	0.126
NBD5	2.1	1.946	0.154
NBD6	2.044	1.901	0.143
NBD7	2.123	1.961	0.162

a Units in eV.

## Open circuit voltage (*V*_oc_) and fill factor (FF)

Open circuit voltage (*V*_oc_) is one of the important parameters to estimate the efficiency of OSCs.^47^ It refers to the maximum voltage produced by the photovoltaic devices to the external circuit when operating at zero current. In OSCs, electricity is produced at the donor of HOMO, transferring electrons to the acceptor of LUMO.^71^ Hence, the *V*_oc_ value primarily depends on the energy levels of the LUMO and HOMO of acceptor and donor, respectively.^72^ The higher value of HOMO and the lower value of LUMO should be required to attain high device performance.^20^ Moreover, the *V*_oc_ is in direct relation with HOMO–LUMO band gap between the designed molecules and the polymer. The higher the band gap between HOMO and LUMO, higher will be the *V*_oc_ value. In organic photovoltaic (OPV) devices, the open circuit voltage (*V*_oc_) is influenced by the HOMO–LUMO band gap of the donor and acceptor materials. As the band gap decreases, the energy difference between the donor's HOMO and the acceptor's LUMO increases, potentially raising the *V*_oc_. This reduction in band gap also allows absorption of a broader spectrum of sunlight, potentially increasing the short circuit current density (*J*_sc_) by generating more charge carriers.^73^

Furthermore, the HOMO/LUMO energy gap of the acceptor and donor units directly increases the PCE values. In this study, the PC_71_BM acceptor polymer is chosen for the calculation of *V*_oc_ of the donor-type designed compounds owing to its confirmed effective charge transference from the donor to acceptors. The *V*_oc_ of the reference compound (NBR) and designed derivatives (NBD1–NBD7) is computed by using the Scharber's Equation.^18^ According to this eqn (2), difference between the HOMO of donor (NBD1–NBD7) and the LUMO of acceptor (PC_71_BM) denotes the *V*_oc_^20^ subtracting 0.3 (an empirical factor).The *V*_oc_ values of all the titled molecules are presented in the Table 5, while their visual representation is displayed in the Fig. 7.2
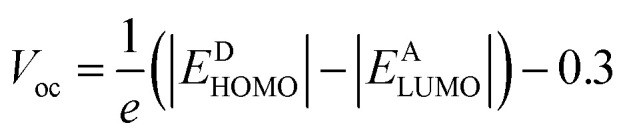


**Table 5:** Open-circuit voltage (*V*_oc_) of the entitled compounds^a^

Compounds	*V*_oc_ (V)	Δ*E*
NBR	2.109	1.959
NBD1	2.143	2.443
NBD2	2.176	2.476
NBD3	2.329	2.629
NBD4	2.347	2.647
NBD5	2.257	2.557
NBD6	2.348	2.648
NBD7	2.178	2.478

a Δ*E* = *E*^A^_LUMO_ − *E*^D^_HOMO_.

**Fig. 7:**
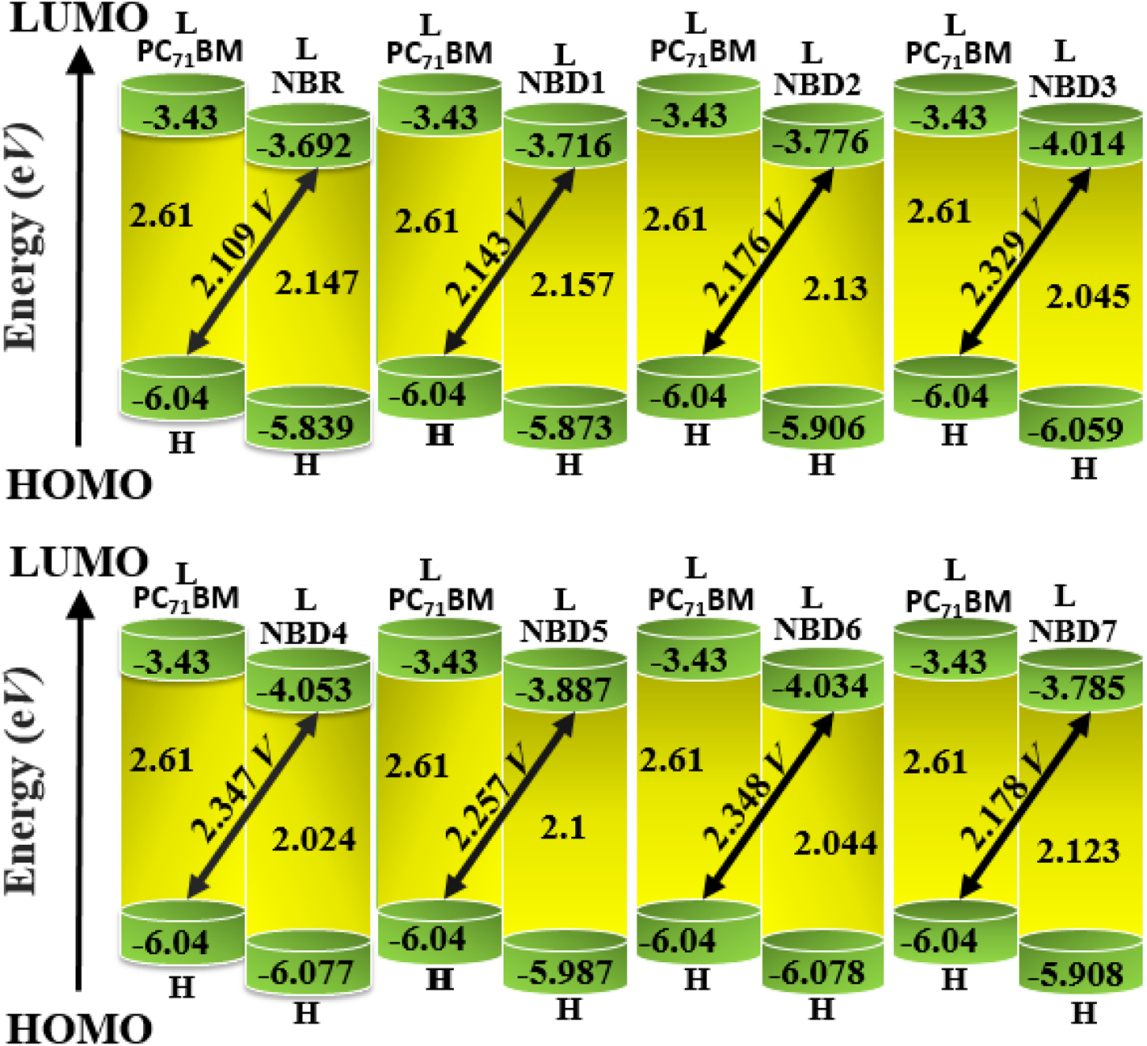
Graphical representation of *V*_oc_ for designed molecules with PC_71_BM.

The results demonstrate that all the tailored compounds (NBD1–NBD7) exhibit greater *V*_oc_ values as compared to NBR reference, owing to their higher HOMO energy values. Notably, NBD6 and NBD4 exhibit the highest *V*_oc_ values of 2.348 and 2.347 V, respectively due to efficient terminal acceptor moieties with planar geometries which allow the ultimate charge transference from donor to acceptor and enhance the conjugation. Therefore, NBD4 emerges as the optimal choice for solar cell applications. Its electron-pulling acceptor group, enhanced by the highly electronegative –NO_2_ attachment, results in excellent photophysical, electronic, and photovoltaic properties compared to all other derivatives. A decreasing order of *V*_oc_ values for all the studied compounds in *V* is as follows: NBD6 (2.348) > NBD4 (2.347) > NBD3 (2.329) > NBD5 (2.257) > NBD7 (2.178) > NBD2 (2.176) > NBD1 (2.143) > NBR (2.109).

Fill factor (FF) significantly influences PCE of organic photovoltaic (PV) devices.^74^ It primarily relies on the open-circuit voltage value.^75^ Higher *V*_oc_ values enhance the fill factor, significantly contributing to the system's efficiency.^76^ It can be computed by using the following eqn (3).^77^3
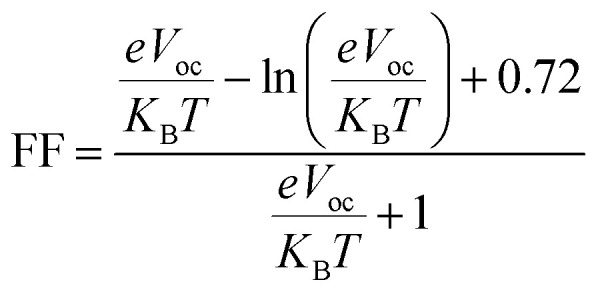
where *K*_B_, *T*, and *e* represent the Boltzmann's constant (8.61733034 × 10^5^), temperature (298 K), and the elementary charge (fixed at 1), respectively. The computed values listed in Table S13† reveal high FF indicating promising PCE.

## Conclusion

In conclusion, a quantum chemical study is performed on the newly designed naphthalene-based molecules (NBD1–NBD7) to explore their optoelectronic, photo-physical and photovoltaic properties. It is noteworthy to discuss that the molecular engineering is performed by incorporating the efficient electron-withdrawing units in NBR which significantly improves the photovoltaic characteristics of the designed derivatives (NBD1–NBD7). They showed reduced energy gap (2.024–2.157 eV) and broadened optical absorption in chloroform (671.087–717.164 nm) and gas phase (623.251–653.404 nm). Further, all derivatives showed higher *V*_oc_ values compared to NBR which are calculated *via* the HOMO_donor_ and polymer LUMO_**PC**_**71**_**BM**_. Exploring the photovoltaic properties more particularly, it is reported that NBD4 is potent among all the designed molecules as it exhibits the maximum absorbance at 717.164 nm (chloroform solvent) and 653.404 nm (gas phase) with lowest binding energy value of 0.126 eV. Moreover, the efficient charge transfer from the HOMO of naphthalene-based donor to the LUMO of the end-capped acceptor units is demonstrated *via* the results of FMOs, DOS and TDM analyses. The results indicate that the designed NFAs-based compounds (NBD1–NBD7) are potential candidates for the next-generation photovoltaic devices.

## Data availability

All data generated or analyzed during this study are included in this published article and its ESI files.†

## Conflicts of interest

There are no conflicts to declare.

## Supplementary Material

RA-014-D4RA03170A-s001
