# Supplementary material for: Unveiling the influence of end-capped acceptors modification on photovoltaic properties of non-fullerene fused ring compounds: a DFT/TD-DFT study
Source: RSC Adv. 2024 Jun 27;14(29):20441–53. doi: 10.1039/d4ra03170a (PMC11208900; doi:10.1039/d4ra03170a)
Supplement: RA-014-D4RA03170A-s001 [file RA-014-D4RA03170A-s001.pdf]

Electronic Supplementary Material (ESI) for RSC Advances.
This journal is © The Royal Society of Chemistry 2024

Supplementary Data

Unveiling the Influence of End-capped Acceptors Modification on Photovoltaic Properties of Non-Fullerene Fused Ring Compounds: A DFT/TD-DFT Study

Muhammad Khalid,[a,b*] Noor Fatima,[a,b] Muhammad Arshad,[c] Muhammad Adeel,[d] Ataualpa A. C. Braga,[e] Tansir Ahamad,[f]

[a]Institute of Chemistry, Khwaja Fareed University of Engineering & Information Technology, Rahim Yar Khan, 64200, Pakistan

[b]Centre for Theoretical and Computational Research, Khwaja Fareed University of Engineering & Information Technology, Rahim Yar Khan, 64200, Pakistan

[c]Industry Solutions, Northern Alberta Institute of Technology, Edmonton, Alberta, Canada

[d]Institute of Chemical Sciences, Gomal University, D.I.Khan, Pakistan.

[e]Departamento de Química Fundamental, Instituto de Química, Universidade de São Paulo, Av. Prof. Lineu Prestes, 748, São Paulo, 05508-000, Brazil

[f]Department of Chemistry, College of Science, King Saud University, Saudi Arabia

*Corresponding author E-mail addresses:

Dr. Muhammad Khalid (khalid@iq.usp.br ; muhammad.khalid@kfueit.edu.pk)

Table S1: Cartesian coordinates of **NBR** compound.

Atoms	X-axis	Y-axis	Z-axis
C	-2.142938	-2.010308	-0.132199
C	-0.808943	-2.302666	-0.144345
C	0.157038	-1.256349	-0.051983
C	-0.284834	0.111447	0.054128
C	-1.67033	0.390788	0.065366
H	1.875854	-2.56476	-0.14226
H	-0.456989	-3.326136	-0.223936
C	1.542381	-1.535974	-0.062289
C	0.681328	1.157796	0.14607
H	-2.003207	1.419723	0.146089
C	2.448591	-0.498736	0.028991
H	0.329471	2.181282	0.22545
C	2.015552	0.866009	0.134198
C	3.213744	1.818609	0.221585
C	4.367337	0.824783	0.153653
C	-2.577861	-0.646021	-0.025777
C	-4.490666	-1.966643	-0.150815
C	-3.340517	-2.963559	-0.219656
C	-4.021875	-0.662109	-0.03973

C	-5.877731	-2.087637	-0.173288
C	-6.598491	-0.885998	-0.080699
H	-6.401741	-3.033043	-0.256028
C	3.890973	-0.477776	0.045054
C	5.756169	0.968219	0.178043
C	6.473639	-0.227535	0.086977
H	6.279658	1.909802	0.256427
C	7.853174	-0.544332	0.086994
C	9.003157	0.223538	0.093095
C	9.109599	1.703342	-0.019275
C	10.364927	-0.314467	0.097225
C	10.546552	2.008092	-0.186035
C	11.293243	0.821421	-0.138376
H	8.029096	-1.612023	0.058062
C	12.678769	0.875957	-0.293136
C	13.246282	2.129827	-0.48628
C	12.51685	3.314552	-0.527288
C	11.135499	3.250341	-0.37316
H	13.324342	0.011877	-0.276949
H	13.034759	4.252784	-0.679208
H	10.525766	4.145008	-0.399417
C	10.772657	-1.613999	0.334146
C	9.900346	-2.687379	0.677313
N	9.242896	-3.593748	0.969836
C	12.135438	-2.028339	0.297607
N	13.224254	-2.418901	0.276737
C	3.220922	2.599146	1.553748
H	3.197276	1.920089	2.408799
H	4.119939	3.216257	1.628111
H	2.351433	3.258574	1.613349
C	3.247271	2.797847	-0.972134
H	2.38166	3.464475	-0.943917
H	4.149617	3.413414	-0.933979
H	3.236892	2.260488	-1.922873
C	-3.3722	-3.943288	0.973681
H	-4.272627	-4.561873	0.935456
H	-2.505148	-4.608088	0.945904
H	-3.363568	-3.406213	1.924592
C	-3.346753	-3.743914	-1.551892
H	-3.324238	-3.064637	-2.406812
H	-2.476537	-4.402357	-1.612133
H	-4.244619	-4.362846	-1.626884
O	8.219172	2.53828	0.000109

F	14.583108	2.198757	-0.642807
S	-5.391177	0.606955	0.052214
S	5.262742	-1.725802	-0.03732
C	-8.010976	-0.905382	-0.092701
H	-8.370759	-1.923623	-0.177569
C	-8.984705	0.070919	-0.0232
C	-10.430758	-0.135329	-0.052439
C	-8.738933	1.52272	0.086105
C	-11.066759	1.207393	0.028001
C	-10.058937	2.18195	0.111772
C	-12.40378	1.603566	0.031003
C	-12.66214	2.966868	0.119038
H	-13.237685	0.922011	-0.030874
C	-10.340353	3.537599	0.199812
C	-11.672283	3.941116	0.203716
H	-9.53603	4.260073	0.263337
H	-11.956012	4.983531	0.269885
C	-11.130418	-1.324589	-0.133509
C	-12.552876	-1.401096	-0.153916
N	-13.703429	-1.520725	-0.175179
C	-10.52643	-2.613069	-0.203835
N	-10.088776	-3.682778	-0.26242
O	-7.656332	2.090688	0.144321
F	-13.949869	3.363932	0.122421

Table S2: Cartesian coordinates of **NBD1** compound.

Atoms	X-axis	Y-axis	Z-axis
C	-2.160013	-2.180351	-0.139343
C	-0.825699	-2.47144	-0.147357
C	0.138902	-1.424415	-0.048407
C	-0.304583	-0.05726	0.060033
C	-1.690264	0.220766	0.067285
H	1.859378	-2.73077	-0.136311
H	-0.472471	-3.494311	-0.228701
C	1.524521	-1.702583	-0.054664
C	0.660026	0.989897	0.157966
H	-2.024416	1.249122	0.149845
C	2.429292	-0.664517	0.042403
H	0.30681	2.01278	0.23875
C	1.99454	0.699544	0.149651
C	3.19118	1.653356	0.243084
C	4.346057	0.66107	0.175918

C	-2.59633	-0.816741	-0.030237
C	-4.507791	-2.138544	-0.16654
C	-3.356501	-3.134278	-0.234538
C	-4.040085	-0.833867	-0.049412
C	-5.894411	-2.260127	-0.194278
C	-6.616216	-1.058849	-0.099972
H	-6.417725	-3.205436	-0.282147
C	3.871533	-0.642145	0.063054
C	5.734239	0.806086	0.203863
C	6.453643	-0.388951	0.112061
H	6.255369	1.748571	0.285614
C	7.832966	-0.703775	0.114316
C	8.982142	0.066158	0.124417
C	9.083486	1.543999	0.017897
C	10.346188	-0.468696	0.130198
C	10.52353	1.852932	-0.138661
C	11.271866	0.669531	-0.090739
H	8.010888	-1.771035	0.084103
C	12.660944	0.732569	-0.232928
C	13.235892	1.979554	-0.415831
C	12.473323	3.151098	-0.454196
C	11.098167	3.102304	-0.31465
H	13.310966	-0.128575	-0.214756
H	10.502953	4.006146	-0.344445
C	10.755649	-1.770083	0.353758
C	9.885128	-2.850576	0.677957
N	9.229272	-3.763084	0.954431
C	12.120266	-2.178057	0.319024
N	13.212272	-2.55963	0.298111
C	3.19352	2.430252	1.577422
H	3.168127	1.748879	2.430567
H	4.091519	3.048278	1.656105
H	2.323075	3.088462	1.636145
C	3.226949	2.635822	-0.947924
H	2.360421	3.301248	-0.920317
H	4.128335	3.252493	-0.905518
H	3.219955	2.101076	-1.900159
C	-3.391868	-4.118198	0.955236
H	-4.291624	-4.73737	0.911497
H	-2.524192	-4.782184	0.928194
H	-3.387119	-3.584547	1.908093
C	-3.357157	-3.909871	-1.569587
H	-3.331827	-3.227581	-2.422013

H	-2.486247	-4.567464	-1.628738
H	-4.254264	-4.529165	-1.650172
O	8.195551	2.380581	0.033932
F	14.564461	2.083775	-0.562082
S	-5.409431	0.43416	0.042259
S	5.244661	-1.888358	-0.01837
C	-8.028029	-1.078602	-0.116481
H	-8.387963	-2.096344	-0.20635
C	-9.002402	-0.102358	-0.045754
C	-10.44935	-0.308437	-0.079269
C	-8.756228	1.346318	0.071174
C	-11.085289	1.031416	0.007271
C	-10.079705	2.004907	0.098063
C	-12.423994	1.430867	0.010178
C	-12.694763	2.78664	0.104813
H	-13.259049	0.750644	-0.057084
C	-10.34922	3.361407	0.19366
C	-11.679025	3.742797	0.196077
H	-9.559402	4.0986	0.263501
C	-11.147937	-1.497632	-0.168574
C	-12.570387	-1.571862	-0.189796
N	-13.721611	-1.684935	-0.210405
C	-10.543797	-2.785487	-0.246487
N	-10.106029	-3.854727	-0.311643
O	-7.677789	1.919803	0.134633
F	-13.966484	3.210523	0.110635
F	13.104033	4.319438	-0.631402
F	-12.021587	5.034621	0.285419

Table S3: Cartesian coordinates of **NBD2** compound.

Atoms	X-axis	Y-axis	Z-axis
C	-2.178446	-2.453772	-0.159041
C	-0.842762	-2.738344	-0.165162
C	0.11629	-1.687583	-0.052322
C	-0.33413	-0.323554	0.068426
C	-1.720931	-0.052248	0.073146
H	1.843369	-2.984512	-0.147351
H	-0.484268	-3.758616	-0.255171
C	1.50308	-1.958933	-0.056354
C	0.62482	0.727303	0.18112
H	-2.06043	0.973556	0.164694
C	2.402355	-0.917311	0.055102

H	0.266282	1.747522	0.271097
C	1.960675	0.443489	0.175336
C	3.152052	1.402015	0.284385
C	4.312	0.416189	0.211468
C	-2.621528	-1.093313	-0.038141
C	-4.526242	-2.422912	-0.194068
C	-3.369945	-3.412414	-0.267601
C	-4.064739	-1.116534	-0.062713
C	-5.911394	-2.55021	-0.227707
C	-6.639188	-1.35214	-0.124796
H	-6.430581	-3.496746	-0.325925
C	3.844062	-0.888646	0.081754
C	5.698524	0.567183	0.247624
C	6.424379	-0.624168	0.145452
H	6.214942	1.510977	0.342632
C	7.803785	-0.932032	0.151165
C	8.950251	-0.155748	0.178354
C	9.043034	1.323001	0.08937
C	10.315723	-0.685056	0.189024
C	10.484158	1.640452	-0.053559
C	11.237865	0.461342	-0.014028
H	7.987855	-1.997805	0.107381
C	12.622603	0.539183	-0.149637
C	13.214002	1.79263	-0.317194
C	12.441694	2.965277	-0.345759
C	11.057571	2.887482	-0.211847
H	13.262133	-0.328764	-0.136417
H	10.446307	3.78027	-0.23301
C	10.730363	-1.986041	0.404897
C	9.862179	-3.073115	0.713072
N	9.207677	-3.990384	0.976589
C	12.097112	-2.387335	0.378524
N	13.190744	-2.764493	0.364267
C	3.144775	2.164304	1.627123
H	3.118912	1.473605	2.472711
H	4.039361	2.785765	1.716486
H	2.270892	2.817611	1.689046
C	3.188102	2.397462	-0.895832
H	2.317856	3.057839	-0.864924
H	4.085905	3.018466	-0.842604
H	3.18829	1.873086	-1.853833
C	-3.404561	-4.407812	0.912638
H	-4.300803	-5.031309	0.859645

H	-2.533242	-5.066839	0.882367
H	-3.406099	-3.883346	1.870582
C	-3.362401	-4.175045	-1.610091
H	-3.337408	-3.484481	-2.455827
H	-2.488163	-4.827871	-1.672507
H	-4.256236	-4.797777	-1.699686
O	8.151381	2.155348	0.107996
S	-5.43893500	0.144877	0.035495
S	5.22256400	-2.127601	-0.007968
C	-8.049444	-1.377967	-0.145753
H	-8.40526	-2.396283	-0.244954
C	-9.029134	-0.405168	-0.070954
C	-10.47436	-0.61796	-0.110675
C	-8.786963	1.042654	0.053647
C	-11.115695	0.721077	-0.024925
C	-10.114372	1.696709	0.074757
C	-12.449732	1.121521	-0.029948
C	-12.74618	2.482882	0.065919
H	-13.269857	0.42561	-0.105833
C	-10.394295	3.046122	0.171671
C	-11.729022	3.445313	0.167641
H	-9.597683	3.774665	0.248116
C	-11.168448	-1.809583	-0.20239
C	-12.59044	-1.890025	-0.228853
N	-13.74094	-2.009307	-0.253822
C	-10.558532	-3.094949	-0.276422
N	-10.115284	-4.162096	-0.338369
O	-7.711246	1.61997	0.125956
Cl	14.94822700	1.860421	-0.490976
Cl	13.17845600	4.531464	-0.549031
Cl	-12.09710200	5.144345	0.289047
Cl	-14.42507100	2.954791	0.055079

Table S4: Cartesian coordinates of **NBD3** compound.

Atoms	X-axis	Y-axis	Z-axis
C	-2.169418	-2.355205	-0.154472
C	-0.83422	-2.641065	-0.160891
C	0.125385	-1.591224	-0.04426
C	-0.323088	-0.226494	0.081334
C	-1.70902	0.046239	0.086691
H	1.850636	-2.889466	-0.144753
H	-0.476398	-3.661144	-0.25442

C	1.511214	-1.864034	-0.049962
C	0.63657	0.82345	0.197265
H	-2.047731	1.071911	0.181527
C	2.411426	-0.822892	0.06424
H	0.2788	1.843515	0.290739
C	1.971999	0.53836	0.189505
C	3.164369	1.495238	0.300007
C	4.322912	0.508331	0.22176
C	-2.610471	-0.994286	-0.028986
C	-4.516534	-2.321752	-0.192966
C	-3.361445	-3.312258	-0.267547
C	-4.052653	-1.014303	-0.055508
C	-5.899279	-2.446179	-0.230453
C	-6.62623	-1.244689	-0.124219
H	-6.420642	-3.390863	-0.333998
C	3.85211	-0.79766	0.088488
C	5.707373	0.656059	0.254647
C	6.431515	-0.539029	0.145775
H	6.225697	1.598589	0.351222
C	7.80629	-0.847816	0.144749
C	8.956488	-0.069453	0.167078
C	9.046143	1.404093	0.071365
C	10.318154	-0.599952	0.175276
C	10.49065	1.724199	-0.082683
C	11.243996	0.545304	-0.04308
H	7.991386	-1.91322	0.096412
C	12.627513	0.617375	-0.195135
C	13.22253	1.873014	-0.377767
C	12.443949	3.056038	-0.402496
C	11.056007	2.973248	-0.252405
H	13.262323	-0.254269	-0.184483
H	10.437562	3.861239	-0.271251
C	10.736964	-1.896704	0.40425
C	9.869191	-2.978397	0.733067
N	9.213725	-3.889586	1.013675
C	12.103338	-2.299198	0.373443
N	13.196628	-2.676648	0.356094
C	3.160248	2.252748	1.645523
H	3.135618	1.559351	2.488893
H	4.055143	2.87362	1.735012
H	2.286831	2.906184	1.710999
C	3.19981	2.494346	-0.877239
H	2.330138	3.155158	-0.842729

H	4.09798	3.114596	-0.822973
H	3.198247	1.973257	-1.836994
C	-3.399291	-4.310379	0.910439
H	-4.295905	-4.932907	0.854105
H	-2.528541	-4.969995	0.879718
H	-3.401916	-3.788422	1.869713
C	-3.352973	-4.071184	-1.612151
H	-3.326247	-3.37862	-2.456151
H	-2.479213	-4.724491	-1.674762
H	-4.247053	-4.693061	-1.704245
O	8.160606	2.242023	0.089402
S	5.42196400	0.249774	0.045299
S	5.22517000	-2.039661	-0.008973
C	-8.032239	-1.268409	-0.149711
H	-8.391258	-2.284882	-0.255348
C	-9.012953	-0.290897	-0.072649
C	-10.455351	-0.501657	-0.117963
C	-8.765496	1.150401	0.061702
C	-11.097727	0.840362	-0.02529
C	-10.094396	1.812348	0.084537
C	-12.432029	1.240584	-0.032504
C	-12.729255	2.606422	0.07377
H	-13.25006	0.543118	-0.117186
C	-10.36356	3.163052	0.191767
C	-11.700856	3.573093	0.18766
H	-9.559825	3.882936	0.276194
C	-11.155628	-1.688327	-0.218423
C	-12.577872	-1.764751	-0.248281
N	-13.72843	-1.881045	-0.276167
C	-10.549061	-2.975191	-0.298709
N	-10.10744	-4.042437	-0.365959
O	-7.692666	1.730854	0.140739
C	13.06287	4.331206	-0.582654
N	13.548636	5.368522	-0.727025
C	14.641272	1.944918	-0.541525
N	15.786991	1.994495	-0.673518
C	-12.016469	4.962093	0.298244
N	-12.255866	6.087841	0.388783
C	-14.099554	3.01511	0.065641
N	-15.208161	3.336786	0.058229

Table S5: Cartesian coordinates of **NBD4** compound.

Atoms	X-axis	Y-axis	Z-axis
C	2.187009	-2.639274	0.179363
C	0.850609	-2.919614	0.17892
C	-0.103667	-1.867346	0.04154
C	0.351497	-0.506079	-0.097554
C	1.738433	-0.239098	-0.095863
H	-1.835232	-3.156937	0.144512
H	0.487878	-3.93699	0.282268
C	-1.490642	-2.134225	0.039706
C	-0.602582	0.546428	-0.233783
H	2.082154	0.783868	-0.201101
C	-2.385451	-1.090662	-0.094269
H	-0.239764	1.563772	-0.336706
C	-1.939172	0.267084	-0.232847
C	-3.126158	1.227793	-0.364002
C	-4.289668	0.247302	-0.280852
C	2.634491	-1.28188	0.039667
C	4.534107	-2.61482	0.233214
C	3.374308	-3.59945	0.313926
C	4.076162	-1.307233	0.075975
C	5.915944	-2.743838	0.279849
C	6.648071	-1.546184	0.161928
H	6.433383	-3.688985	0.397759
C	-3.825767	-1.059457	-0.129148
C	-5.672901	0.401004	-0.322895
C	-6.403369	-0.789576	-0.20378
H	-6.185776	1.344988	-0.43278
C	-7.779341	-1.09031	-0.206164
C	-8.924511	-0.304059	-0.235552
C	-9.004505	1.169495	-0.13859
C	-10.288895	-0.826169	-0.246696
C	-10.448268	1.497518	0.012937
C	-11.207299	0.324558	-0.026091
H	-7.972008	-2.154208	-0.152313
C	-12.593071	0.405982	0.119922
C	-13.162349	1.660235	0.302076
C	-12.385361	2.822285	0.34521
C	-11.008993	2.749975	0.191327
H	-13.246572	-0.45176	0.096164
H	-10.398447	3.642305	0.230334
C	-10.717338	-2.118609	-0.479065
C	-9.856835	-3.205044	-0.81152
N	-9.206854	-4.119083	-1.095248

C	-12.087127	-2.509467	-0.448108
N	-13.184288	-2.8753	-0.430877
C	-3.109383	1.967224	-1.719521
H	-3.082239	1.262491	-2.553361
H	-4.000703	2.590943	-1.823447
H	-2.232488	2.615688	-1.787746
C	-3.164732	2.242663	0.799559
H	-2.29135	2.8984	0.762306
H	-4.059186	2.866798	0.730719
H	-3.172592	1.734491	1.766185
C	3.415667	-4.614682	-0.849158
H	4.308777	-5.24064	-0.777481
H	2.541488	-5.269566	-0.814958
H	3.427531	-4.106796	-1.815883
C	3.353684	-4.338696	1.669405
H	3.324131	-3.633793	2.503031
H	2.476822	-4.987464	1.735631
H	4.244555	-4.962723	1.776604
O	-8.11551	2.0032	-0.153098
Se	5.450056	-0.049485	-0.032657
Se	-5.204948	-2.293986	-0.025747
C	8.053412	-1.574423	0.192198
H	8.409414	-2.590516	0.311388
C	9.03758	-0.600515	0.106253
C	10.478834	-0.815766	0.156153
C	8.794762	0.839929	-0.04006
C	11.125133	0.524034	0.059201
C	10.126915	1.496698	-0.064852
C	12.462645	0.92123	0.064936
C	12.747254	2.276578	-0.051282
H	13.288964	0.232898	0.147197
C	10.403352	2.847986	-0.175592
C	11.735276	3.234928	-0.161101
H	9.61337	3.583086	-0.253854
C	11.177422	-2.002378	0.26172
C	12.59967	-2.078123	0.295364
N	13.750344	-2.191988	0.325485
C	10.568789	-3.288255	0.34234
N	10.124547	-4.354312	0.409741
O	7.725548	1.425153	-0.125877
N	-12.966731	4.144257	0.659886
O	-13.811161	4.186034	1.539127
O	-12.521074	5.101278	0.048384

N	-14.640763	1.717462	0.346196
O	-15.17969	2.603375	-0.296108
O	-15.209945	0.848444	0.985159
N	12.023308	4.684607	-0.162577
O	12.873643	5.084201	0.615228
O	11.35217	5.378042	-0.908989
N	14.176305	2.647746	-0.156387
O	14.955441	2.065172	0.578917
O	14.471989	3.47645	-1.001286

Table S6: Cartesian coordinates of **NBD5** compound.

Atoms	X-axis	Y-axis	Z-axis
C	2.205403	-2.894874	0.183334
C	0.86798	-3.170565	0.184852
C	-0.082953	-2.115474	0.046966
C	0.377057	-0.756394	-0.094717
C	1.765359	-0.494258	-0.094345
H	-1.818884	-3.399241	0.153352
H	0.502039	-4.186678	0.289867
C	-1.47113	-2.377759	0.046522
C	-0.573966	0.29871	-0.232667
H	2.112438	0.527449	-0.201303
C	-2.362575	-1.332054	-0.089319
H	-0.208095	1.314788	-0.337905
C	-1.911604	0.023643	-0.231113
C	-3.095897	0.987586	-0.365266
C	-4.262677	0.010749	-0.281356
C	2.658071	-1.539505	0.04171
C	4.552694	-2.879175	0.234603
C	3.38929	-3.859364	0.317215
C	4.100424	-1.571123	0.076923
C	5.935376	-3.014473	0.280266
C	6.672091	-1.821653	0.161728
H	6.448241	-3.962145	0.398286
C	-3.803318	-1.295545	-0.125036
C	-5.646866	0.168721	-0.327799
C	-6.380594	-1.017798	-0.20713
H	-6.157215	1.113532	-0.443067
C	-7.759566	-1.316558	-0.21242
C	-8.902245	-0.531688	-0.256899
C	-8.984652	0.94669	-0.192283
C	-10.269216	-1.052186	-0.263075

C	-10.427354	1.27536	-0.053976
C	-11.184976	0.105629	-0.074134
H	-7.952207	-2.37989	-0.148562
C	-12.568501	0.19948	0.070633
C	-13.166213	1.452757	0.227937
C	-12.383715	2.629081	0.222756
C	-10.999866	2.525763	0.085163
H	-13.199931	-0.67306	0.07678
H	-10.374589	3.407721	0.084795
C	-10.696183	-2.349802	-0.468123
C	-9.835265	-3.446374	-0.76456
N	-9.185649	-4.369652	-1.018125
C	-12.066569	-2.739817	-0.442895
N	-13.162144	-3.11082	-0.430239
C	-3.075463	1.724685	-1.721927
H	-3.049174	1.018312	-2.554433
H	-3.965151	2.350412	-1.828186
H	-2.196817	2.370829	-1.790501
C	-3.132466	2.004816	0.79627
H	-2.256909	2.657734	0.75901
H	-4.024955	2.631617	0.725411
H	-3.143089	1.498347	1.763788
C	3.425231	-4.875527	-0.845211
H	4.315975	-5.505024	-0.774317
H	2.548443	-5.526939	-0.809933
H	3.438146	-4.368128	-1.812205
C	3.367302	-4.597984	1.672971
H	3.341167	-3.892464	2.506215
H	2.488202	-5.243648	1.740674
H	4.256125	-5.225078	1.779731
O	-8.091524	1.776239	-0.223517
S	5.48175800	-0.319922	-0.033219
S	-5.18787900	-2.52503	-0.019898
C	8.079643	-1.856153	0.192511
H	8.429494	-2.874518	0.310829
C	9.067108	-0.888755	0.109605
C	10.508959	-1.10992	0.162189
C	8.833732	0.556512	-0.034322
C	11.158784	0.228264	0.07298
C	10.168462	1.203823	-0.047561
C	12.493614	0.628122	0.095053
C	12.814131	1.985086	-0.005278
H	13.297621	-0.081561	0.195601

C	10.463994	2.549574	-0.156518
C	11.798126	2.95649	-0.143345
H	9.665044	3.271394	-0.254114
C	11.199228	-2.301967	0.262085
C	12.621017	-2.389035	0.295998
N	13.77023	-2.5174	0.326929
C	10.583007	-3.584786	0.335062
N	10.133225	-4.649031	0.396285
O	7.764958	1.142785	-0.125258
C	12.059698	4.449982	-0.233868
C	14.295575	2.326665	-0.00609
C	-12.950673	4.025396	0.411609
C	-14.681638	1.463679	0.350798
F	-14.064092	4.238417	-0.314413
F	-13.253238	4.26984	1.703398
F	-12.058579	4.966961	0.039317
F	-15.271167	1.834987	-0.804274
F	-15.114577	2.298098	1.315134
F	-15.161363	0.240282	0.654075
F	13.067784	4.751806	-1.073432
F	14.742874	2.592486	-1.250616
F	14.579453	3.393663	0.76494
F	15.038422	1.303141	0.461685
F	12.359652	4.978118	0.970614
F	10.97081	5.107819	-0.683832

Table S7: Cartesian coordinates of **NBD6** compound.

Atoms	X-axis	Y-axis	Z-axis
C	2.212765	-3.101562	0.19305
C	0.874544	-3.372873	0.189487
C	-0.072342	-2.313589	0.055985
C	0.392017	-0.954568	-0.074389
C	1.780729	-0.696919	-0.069562
H	-1.812314	-3.592799	0.145516
H	0.504924	-4.388355	0.286669
C	-1.460962	-2.571661	0.048213
C	-0.55506	0.104878	-0.206548
H	2.131516	0.324223	-0.168936
C	-2.348587	-1.521809	-0.083094
H	-0.185544	1.120486	-0.302443
C	-1.893418	-0.166035	-0.211471
C	-3.074198	0.802604	-0.341569

C	-4.243912	-0.171732	-0.272478
C	2.669656	-1.746579	0.060647
C	4.559769	-3.0942	0.246505
C	3.393048	-4.070753	0.323516
C	4.111107	-1.782299	0.096249
C	5.940348	-3.233632	0.290879
C	6.681296	-2.04066	0.178004
H	6.450948	-4.183111	0.403686
C	-3.788489	-1.481881	-0.126039
C	-5.626123	-0.010244	-0.324944
C	-6.363775	-1.197672	-0.221205
H	-6.132898	0.937267	-0.432711
C	-7.741157	-1.492523	-0.243303
C	-8.882599	-0.701966	-0.287487
C	-8.958008	0.771719	-0.187528
C	-10.249666	-1.217718	-0.327144
C	-10.403521	1.103801	-0.067497
C	-11.16632	-0.060207	-0.129786
H	-7.938518	-2.555889	-0.19741
C	-12.554554	0.033225	-0.009563
C	-13.142959	1.285312	0.164622
C	-12.351174	2.452599	0.213755
C	-10.966503	2.355824	0.102794
H	-13.202479	-0.829027	-0.038583
H	-10.340714	3.236539	0.161482
C	-10.680008	-2.507821	-0.565251
C	-9.817404	-3.601052	-0.870036
N	-9.165738	-4.52036	-1.13192
C	-12.051656	-2.894432	-0.569021
N	-13.146596	-3.266719	-0.58152
C	-3.045863	1.552666	-1.690978
H	-3.017295	0.854467	-2.530259
H	-3.933573	2.181521	-1.795029
H	-2.165503	2.197444	-1.748985
C	-3.113154	1.808451	0.829821
H	-2.235607	2.459089	0.802537
H	-4.00349	2.438489	0.761057
H	-3.129464	1.292677	1.792296
C	3.426777	-5.081924	-0.843281
H	4.314839	-5.715273	-0.77374
H	2.547407	-5.729941	-0.811668
H	3.44289	-4.570575	-1.808117
C	3.367461	-4.814836	1.676328

H	3.342734	-4.112805	2.51253
H	2.486206	-5.457862	1.740219
H	4.254095	-5.445264	1.781139
O	-8.065384	1.60189	-0.180413
Se	5.494022	-0.534186	-0.008091
Se	-5.175035	-2.709436	-0.04196
C	8.08591	-2.079781	0.207867
H	8.434087	-3.099146	0.321839
C	9.077884	-1.112812	0.12678
C	10.517206	-1.339169	0.17789
C	8.84614	0.329295	-0.015595
C	11.173164	-0.002908	0.081481
C	10.184718	0.97517	-0.0364
C	12.514271	0.38702	0.091283
C	12.820566	1.742078	-0.012204
H	13.328865	-0.315993	0.173902
C	10.472954	2.325139	-0.145178
C	11.807474	2.714585	-0.138084
H	9.681378	3.058548	-0.228665
C	11.207057	-2.530178	0.286811
C	12.628612	-2.618953	0.323449
N	13.777396	-2.749112	0.357528
C	10.588146	-3.811141	0.370571
N	10.135653	-4.873495	0.440816
O	7.78239	0.924162	-0.10135
O	-14.117173	4.089275	1.373519
O	-11.8322	4.978373	0.804633
O	-15.479745	2.228274	-0.681099
O	-15.378185	-0.139875	0.183101
O	13.113164	4.821493	0.789928
O	10.837335	5.186232	-0.221245
O	15.347302	0.901941	-0.011976
O	14.80811	3.099521	-1.113713
O	-15.271265	1.6976	1.766468
O	-13.563786	4.579959	-0.949308
O	12.728389	4.718982	-1.683294
O	14.849704	2.840189	1.37693
H	14.368096	3.702516	1.388794
H	13.610386	4.275534	-1.717888
H	-12.859021	4.955575	-1.503378
H	-15.044449	2.651534	1.855598
S	-14.963183	1.246133	0.255612
S	-12.966072	4.134563	0.480652

S	12.106577	4.497071	-0.22655
S	14.590769	2.131438	-0.033314

Table S8: Cartesian coordinates of **NBD7** compound.

Atoms	X-axis	Y-axis	Z-axis
C	-2.213583	-2.927526	-0.139369
C	-0.87588	-3.202333	-0.135299
C	0.075067	-2.142478	-0.040282
C	-0.385673	-0.779683	0.052431
C	-1.774428	-0.518431	0.045373
H	1.811804	-3.428238	-0.105413
H	-0.509661	-4.221552	-0.203207
C	1.463699	-2.40371	-0.034564
C	0.565168	0.280001	0.150187
H	-2.121752	0.506462	0.114893
C	2.35497	-1.353595	0.060972
H	0.198927	1.299043	0.219979
C	1.903068	0.00585	0.155707
C	3.087064	0.974744	0.253754
C	4.254362	-0.00376	0.202035
C	-2.666999	-1.56805	-0.04887
C	-4.561263	-2.91471	-0.182746
C	-3.397661	-3.897118	-0.230756
C	-4.109939	-1.60229	-0.078651
C	-5.945257	-3.05323	-0.217239
C	-6.682174	-1.858606	-0.143664
H	-6.457005	-4.00582	-0.293399
C	3.796157	-1.314176	0.091438
C	5.639231	0.15764	0.241392
C	6.374161	-1.030231	0.159043
H	6.148573	1.106368	0.324961
C	7.755144	-1.327639	0.167869
C	8.896546	-0.542538	0.190979
C	8.978776	0.93559	0.106284
C	10.26605	-1.062015	0.196747
C	10.418852	1.264699	-0.045711
C	11.179347	0.089743	-0.019356
H	7.947586	-2.392147	0.128409
C	12.558173	0.181795	-0.184306
C	13.151349	1.440447	-0.350482
C	12.371889	2.613905	-0.359913
C	10.984801	2.514631	-0.211533

H	13.20873	-0.678852	-0.199828
H	10.352791	3.394549	-0.237199
C	10.692404	-2.356342	0.424365
C	9.832865	-3.446924	0.745338
N	9.184544	-4.365412	1.019517
C	12.062763	-2.747136	0.40504
N	13.156752	-3.123071	0.401422
C	3.068564	1.759132	1.583583
H	3.042971	1.082455	2.440445
H	3.958839	2.387633	1.66661
H	2.190352	2.40788	1.63045
C	3.121235	1.950689	-0.942669
H	2.245749	2.604611	-0.926689
H	4.013959	2.579471	-0.895557
H	3.130078	1.410505	-1.89179
C	-3.428914	-4.866496	0.971032
H	-4.320334	-5.497688	0.928779
H	-2.552606	-5.519415	0.958042
H	-3.437693	-4.321056	1.917154
C	-3.380429	-4.689081	-1.556039
H	-3.356929	-4.017237	-2.416754
H	-2.501865	-5.337532	-1.601009
H	-4.269972	-5.319387	-1.634697
O	8.082991	1.763474	0.134634
S	-5.49392100	-0.349137	-0.015846
S	5.18334800	-2.544391	0.024213
C	-8.092215	-1.896141	-0.159863
H	-8.440062	-2.919429	-0.232754
C	-9.07908	-0.929114	-0.107495
C	-10.522445	-1.153623	-0.129548
C	-8.848068	0.524551	-0.042612
C	-11.174064	0.184683	-0.113041
C	-10.180166	1.170574	-0.052697
C	-12.511448	0.577427	-0.145495
C	-12.825719	1.939155	-0.100889
H	-13.322355	-0.132233	-0.198157
C	-10.474001	2.521057	-0.029386
C	-11.814312	2.918367	-0.061071
H	-9.680626	3.255796	-0.004433
C	-11.208516	-2.352924	-0.140662
C	-12.630007	-2.449543	-0.153909
N	-13.778323	-2.589211	-0.163482
C	-10.587582	-3.635388	-0.127288

N	-10.134817	-4.700226	-0.114916
O	-7.775759	1.110938	0.006074
O	13.014642	4.372318	-1.811996
O	13.201722	4.819225	0.340863
O	15.165036	2.6612	-0.343546
O	15.292097	0.454895	-0.769755
O	-13.180087	4.786806	-0.676077
O	-11.194043	5.154016	0.31954
O	-15.072347	1.902336	-0.925866
O	-14.62082	2.971847	1.00941
C	16.592408	2.790318	-0.534891
H	16.809636	3.841678	-0.368517
H	17.123003	2.165869	0.183473
H	16.859561	2.496248	-1.549519
C	13.19732	4.3481	1.706784
H	12.203206	4.014324	2.005338
H	13.924274	3.546512	1.8351
H	13.488563	5.208726	2.303745
C	-11.403113	6.578714	0.196131
H	-10.523339	7.037459	0.638848
H	-11.496086	6.855683	-0.854101
H	-12.303922	6.873711	0.734239
C	-15.999486	3.395234	1.102422
H	-16.080832	3.901663	2.060341
H	-16.231459	4.078729	0.285223
H	-16.66465	2.532834	1.063345
C	12.922995	3.989198	-0.673768
C	14.640925	1.441619	-0.519946
C	-12.162542	4.370811	-0.171085
C	-14.292476	2.281381	-0.086291

Table S9: Calculated energy (*E*) and energy gap (ΔE) of HOMO-1/LUMO+1 and HOMO-2/LUMO+2 for the entitled compounds in *eV*.

Compounds	HOMO-1	LUMO+1	ΔE	HOMO-2	LUMO+2	ΔE
**NBR**	-6.540	-2.977	3.077	-6.729	-2.928	3.71
**NBD1**	-6.548	-3.017	3.105	-6.746	-2.945	3.652
**NBD2**	-6.557	-3.057	3.069	-6.782	-2.967	3.594
**NBD3**	-6.561	-3.101	2.908	-6.776	-2.987	3.206
**NBD4**	-6.601	-3.580	2.815	-6.895	-3.081	3.035
**NBD5**	-6.581	-3.287	3.017	-6.844	-3.024	3.418
**NBD6**	-6.602	-4.000	2.898	-6.910	-3.212	3.197
**NBD7**	-6.619	-3.832	3.049	-6.917	-3.131	3.488

Table S10: Wave length, excitation energy and oscillator strength of **NBR** and **NBD1-NBD7** in solvent phase.

Compounds	λ (*nm*)	*E* (*eV*)	f_{os}	MO contributions

**NBR**	565.772	2.191	0.002	H→L+1 (97%)
	493.054	2.515	0.010	H-1→L (88%), H→L+3 (8%)
	488.566	2.538	0.225	H-2→L (90%), H→L+2 (7%)
	485.372	2.554	0.110	H→L+2 (86%), H-2→L (7%), H-1→L+3 (3%)
	458.620	2.703	0.035	H→L+3 (83%), H-1→L (9%), H-1→L+2 (3%)
**NBD1**	561.900	2.207	0.002	H→L+1 (96%)
	495.062	2.504	0.179	H→L+2 (86%), H-1→L (5%), H-1→L+3 (3%)
	491.958	2.520	0.007	H-1→L (71%), H→L+3 (18%), H→L+2 (7%)
	489.028	2.535	0.129	H-2→L (96%)
	467.174	2.654	0.034	H-1→L (21%), H→L+3 (72%)
**NBD2**	568.522	2.181	0.001	H→L+1 (97%)
	503.057	2.465	0.196	H→L+2 (90%), H-1→L (2%), H-1→L+3 (3%)
	498.105	2.489	0.003	H-1→L (72%), H→L+3 (21%), H→L+2 (3%)
	495.695	2.501	0.126	H-2→L (96%)
	475.433	2.608	0.046	H-1→L (23%), H→L+3 (71%)
**NBD3**	618.371	2.005	0.007	H→L+1 (96%)
	577.042	2.149	0.351	H→L+2 (96%), H-1→L+3 (2%)
	534.918	2.318	0.031	H-1→L (22%), H→L+3 (74%)
	518.824	2.390	0.160	H-2→L (96%)
	503.281	2.464	0.012	H-1→L (76%), H→L+3 (21%)
**NBD4**	653.645	1.897	0.022	H→L+1 (96%)
	619.855	2.000	0.583	H→L+2 (97%)
	551.503	2.248	0.025	H-1→L (12%), H→L+3 (85%)
	522.894	2.371	0.162	H-2→L (94%), H-2→L+2 (3%)
	510.240	2.430	0.002	H-1→L (85%), H→L+3 (13%)
**NBD5**	584.551	2.121	0.004	H→L+1 (96%)
	531.935	2.331	0.222	H→L+2 (95%), H-1→L+3 (3%)
	510.409	2.429	0.025	H-1→L (45%), H→L+3 (51%)
	505.931	2.451	0.148	H-2→L (96%)
	487.759	2.542	0.022	H-1→L (53%), H→L+3 (43%)
**NBD6**	623.847	1.987	0.022	H→L+1 (95%)
	578.146	2.145	0.394	H→L+2 (96%), H-1→L+3 (2%)
	532.026	2.330	0.032	H-1→L (27%), H→L+3 (69%)
	520.588	2.382	0.163	H-2→L (96%)
	502.099	2.469	0.011	H-1→L (71%), H→L+3 (27%)
**NBD7**	574.396	2.159	0.014	H→L+1 (96%)
	521.114	2.379	0.146	H→L+2 (94%)
	504.120	2.459	0.046	H-1→L (50%), H→L+3 (47%)
	497.206	2.494	0.152	H-2→L (96%)
	484.102	2.561	0.033	H-1→L (48%), H→L+3 (47%), H→L+1 (2%)

MO=molecular orbital, H=HOMO, L=LUMO, f_{os}= oscillator strength, wavelength= λ (*nm*)

Table S11: Wave length, excitation energy and oscillator strength of **NBR** and **NBD1-NBD7** in gas phase.

**Compounds**	λ **(*nm*)**	***E* (*eV*)**	f_{os}	**MO contributions**
**NBR**	541.412	2.290	0.002	H-1→L (10%), H→L+1 (88%)
	474.777	2.611	0.025	H-1→L (65%), H→L+3 (20%), H→L+1 (4%), H→L+2 (6%)
	468.888	2.644	0.221	H-2→L (13%), H→L+2 (74%), H-1→L (4%), H-1→L+3 (3%)
	464.218	2.671	0.031	H-2→L (83%), H→L+2 (11%)
	441.379	2.809	0.016	H-1→L (20%), H→L+3 (68%), H→L+1 (5%)
	536.841	2.310	0.002	H-1→L (10%), H→L+1 (87%)

**NBD1**	477.962	2.594	0.094	H-1→L (24%), H→L+2 (55%), H→L+3 (13%)
	474.015	2.616	0.058	H-1→L (29%), H→L+2 (35%), H→L+3 (27%)
	465.858	2.661	0.088	H-2→L (95%)
	446.996	2.774	0.014	H-1→L (36%), H→L+3 (50%), H→L+1 (8%)
**NBD2**	541.838	2.288	0.002	H-1→L (11%), H→L+1 (87%)
	483.027	2.567	0.106	H-1→L (20%), H→L+2 (62%), H→L+3 (11%)
	478.700	2.590	0.050	H-1→L (32%), H→L+2 (29%), H→L+3 (30%), H→L+1 (2%)
	470.079	2.638	0.086	H-2→L (94%)
	453.073	2.737	0.022	H-1→L (37%), H→L+3 (49%), H→L+1 (8%)
**NBD3**	573.254	2.163	0.003	H→L+1 (89%), H-1→L (4%), H→L+3 (3%)
	537.352	2.307	0.234	H→L+2 (95%), H-1→L+3 (2%)
	516.877	2.399	0.018	H-1→L (29%), H→L+3 (68%)
	488.835	2.536	0.115	H-2→L (96%)
	471.437	2.630	0.013	H-1→L (65%), H→L+3 (26%), H→L+1 (7%)
**NBD4**	588.100	2.108	0.015	H→L+1 (91%), H-1→L+2 (2%), H→L+3 (4%)
	559.946	2.214	0.307	H→L+2 (95%)
	527.207	2.352	0.017	H-1→L (27%), H→L+3 (69%)
	490.770	2.526	0.120	H-2→L (96%)
	474.106	2.615	0.007	H-1→L (69%), H→L+3 (24%), H→L+1 (4%)
**NBD5**	552.609	2.244	0.002	H→L+1 (88%), H-1→L (8%)
	505.910	2.451	0.166	H→L+2 (93%), H-1→L+3 (2%)
	494.884	2.505	0.020	H-1→L (34%), H→L+3 (60%)
	478.959	2.589	0.104	H-2→L (96%)
	460.494	2.692	0.013	H-1→L (54%), H→L+3 (33%), H→L+1 (9%)
**NBD6**	580.148	2.137	0.035	H→L+1 (90%), H-1→L (3%), H→L+2 (2%), H→L+3 (3%)
	543.834	2.280	0.249	H→L+2 (92%), H-1→L (2%)
	516.619	2.400	0.036	H-1→L (31%), H→L+3 (66%)
	490.130	2.530	0.116	H-2→L (96%)
	471.205	2.631	0.012	H-1→L (62%), H→L+3 (27%), H→L+1 (6%), H→L+2 (2%)
**NBD7**	543.001	2.283	0.000	H-1→L (10%), H→L+1 (87%)
	494.273	2.508	0.080	H→L+2 (93%), H-1→L+2 (2%)
	485.828	2.552	0.074	H-1→L (40%), H→L+3 (54%)
	468.782	2.645	0.098	H-2→L (95%)
	457.486	2.710	0.022	H-1→L (47%), H→L+1 (10%), H→L+3 (39%)

MO=molecular orbital, H=HOMO, L=LUMO, f_{os}= oscillator strength, wavelength= λ (*nm*)

Table S12: Percentages for HOMOs and LUMOs of the studied compounds.

	**LUMO**		**HOMO**	
**Compound**	**Acceptor (%)**	**Donor (%)**	**Acceptor (%)**	**Donor (%)**
**NBR**	61.1	38.9	30.0	70.0
**NBD1**	60.0	40.0	29.7	70.3
**NBD2**	60.5	39.5	30.4	69.6
**NBD3**	65.0	35.0	31.5	68.5
**NBD4**	69.6	30.4	31.5	68.5
**NBD5**	61.7	38.3	30.8	69.2
**NBD6**	65.7	34.3	31.5	68.5
**NBD7**	61.1	38.9	30.5	69.5

Table S13: Open circuit voltage (V_{oc}), Band Gap(**ΔE**), eV_{oc}/K_BT, FF, L_D-$L_A(eV)$ & PCE (%) of entitled compounds.

Compounds	V_{oc}	ΔE	eV_{oc}/K_BT	FF	PCE (%)
**NBR**	2.109	2.147	81.429	0.965	29.86
**NBD1**	2.143	2.157	82.741	0.965	30.34
**NBD2**	2.176	2.130	84.015	0.966	30.84
**NBD3**	2.329	2.045	89.92	0.975	33.31
**NBD4**	2.347	2.024	90.618	0.968	33.33
**NBD5**	2.257	2.100	87.143	0.967	30.02
**NBD6**	2.348	2.044	90.656	0.968	33.34
**NBD7**	2.178	2.123	84.093	0.966	30.86

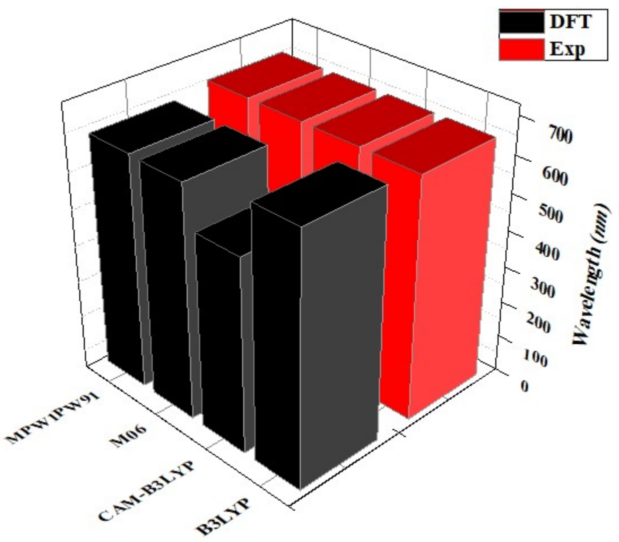

Figure S1: A comparative UV–Vis analysis between experimental and DFT-based data of **NBR** compound in chloroform solvent.

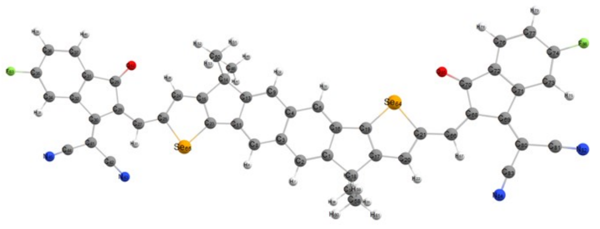

NBR

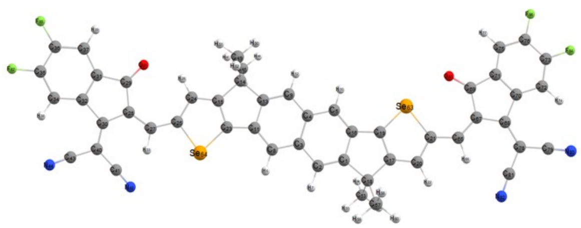

NBD1

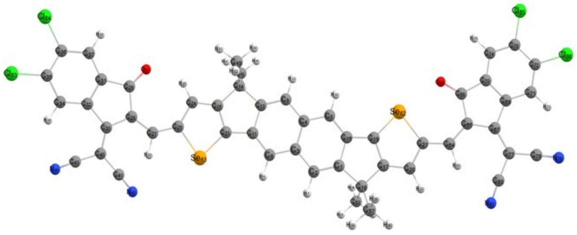

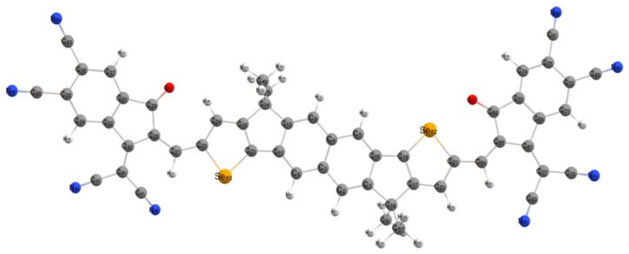

NBD2 **NBD3**

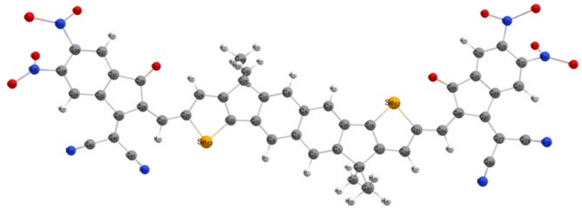

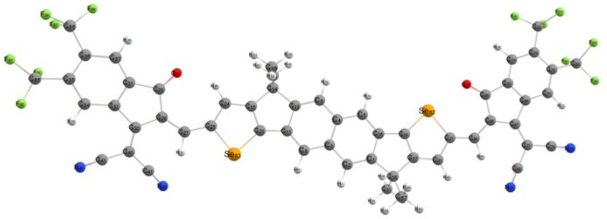

NBD4 **NBD5**

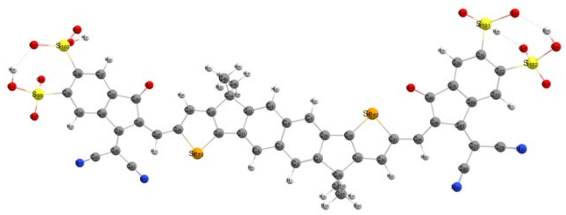

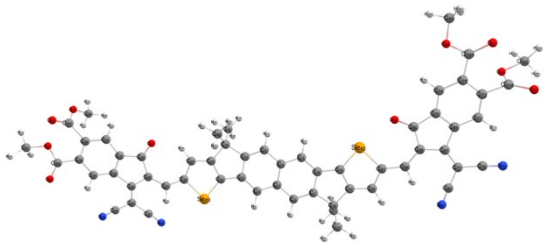

NBD6 **NBD7**

Figure S2: The optimized structures of the investigated compounds **NBR** and **NBD1-NBD7.**

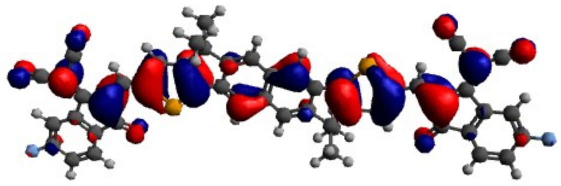

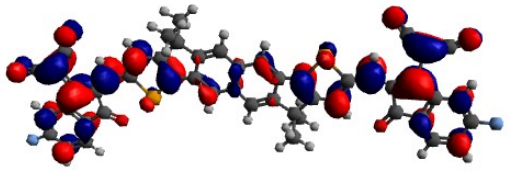

HOMO-1 **LUMO+1**

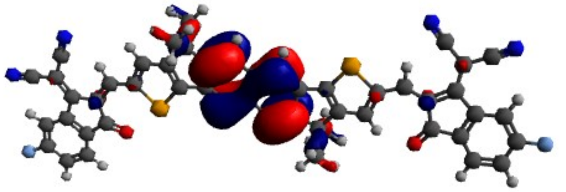

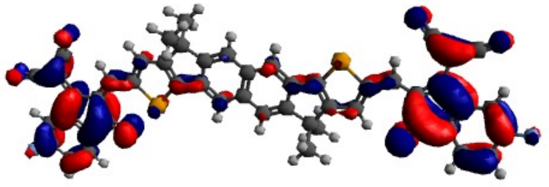

HOMO-2 **LUMO+2**

NBR

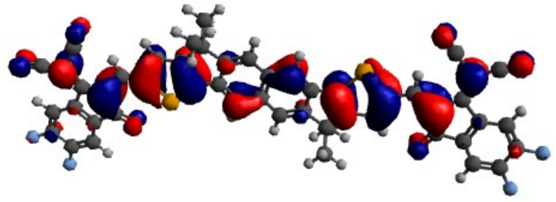

HOMO-1

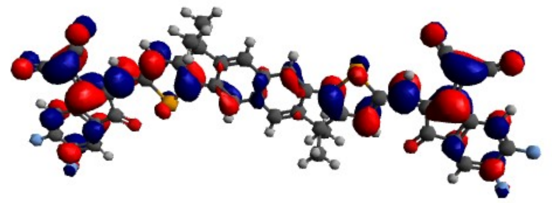

LUMO+1

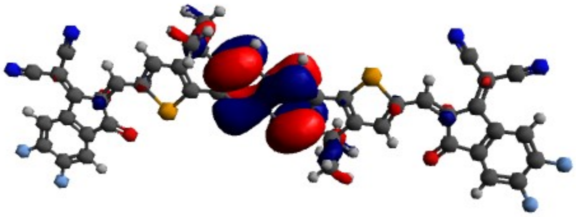

HOMO-2

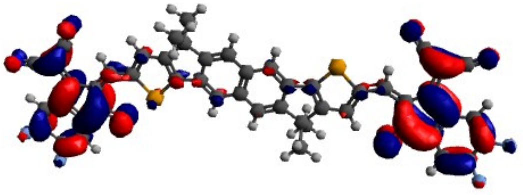

LUMO+2

NBD1

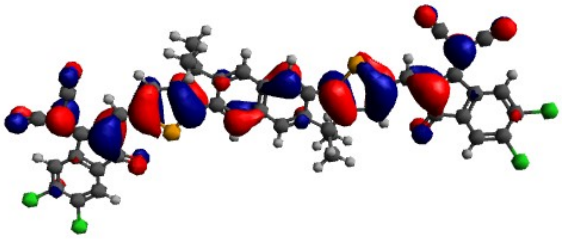

HOMO-1

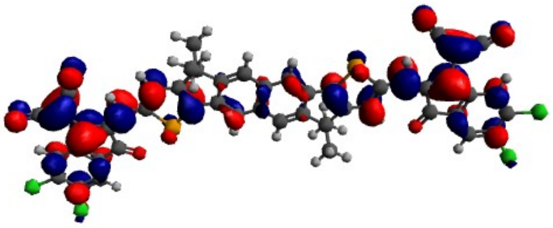

LUMO+1

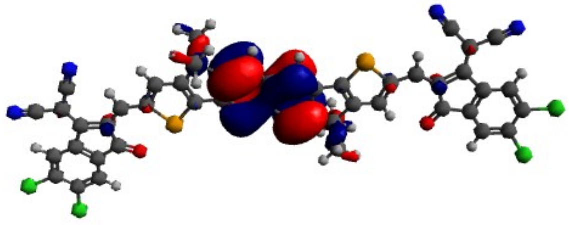

HOMO-2

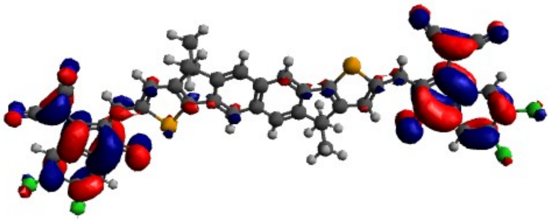

LUMO+2

NBD2

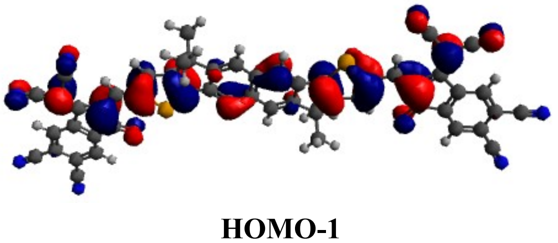

HOMO-1

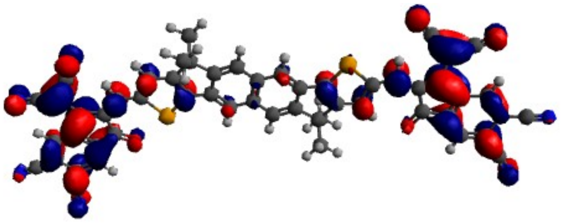

LUMO+1

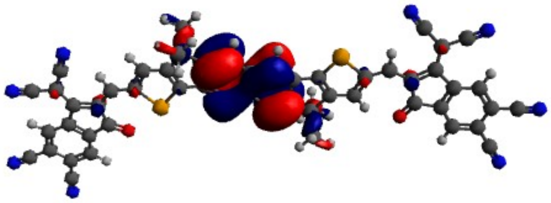

HOMO-2

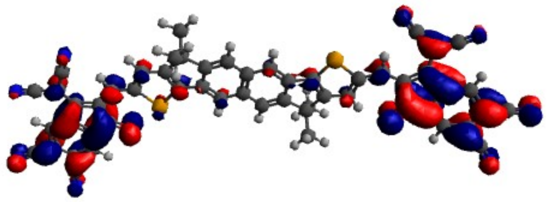

LUMO+2

NBD3

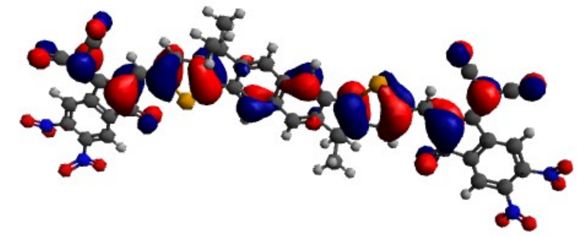

HOMO-1

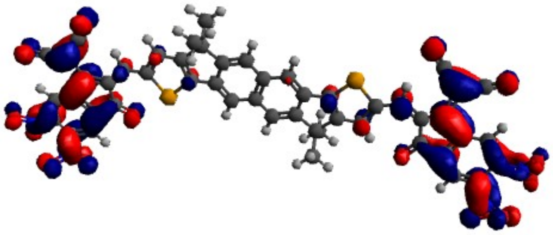

LUMO+1

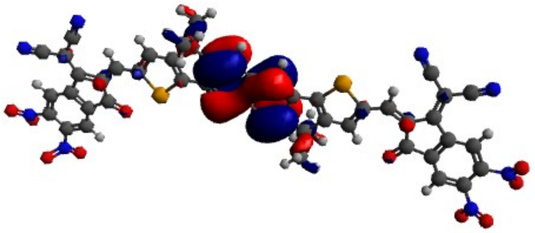

HOMO-2

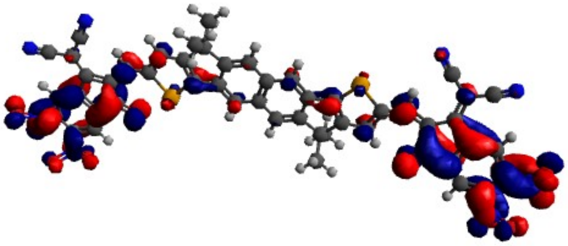

LUMO+2

NBD4

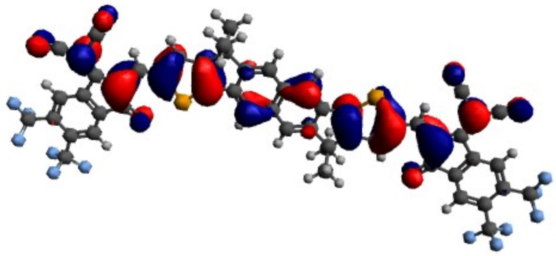

HOMO-1

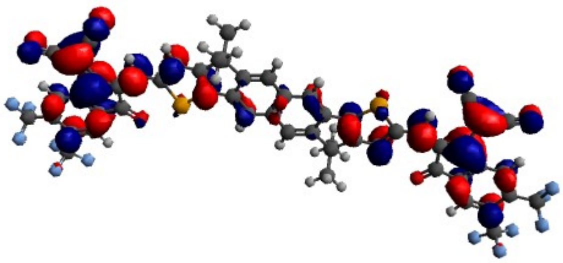

LUMO+1

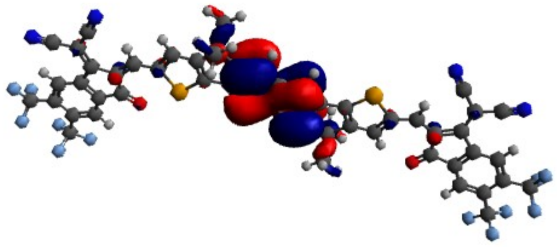

HOMO-2

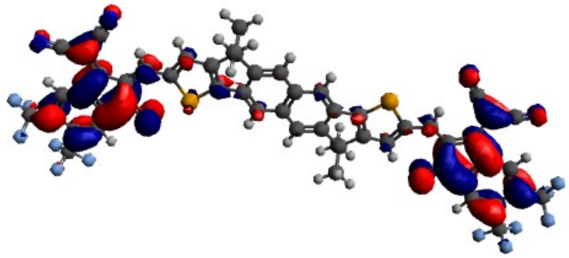

LUMO+2

NBD5

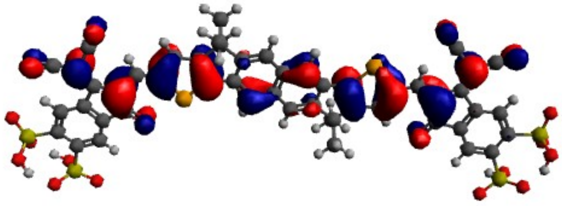

HOMO-1

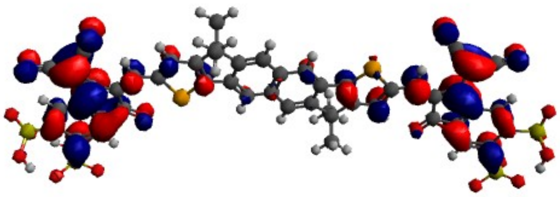

LUMO+1

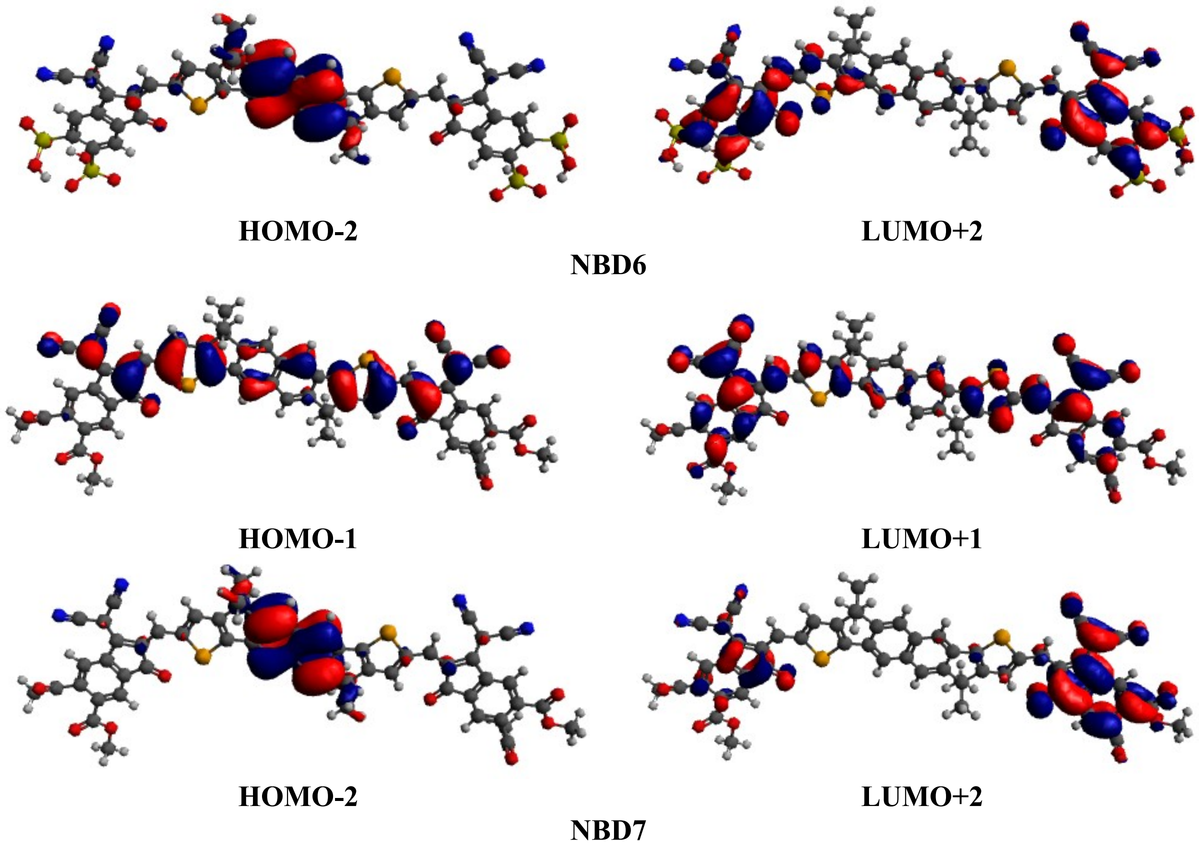

Figure S3: Frontier molecular orbital contour plots of HOMO+1/LUMO-1 and HOMO+2/LUMO-2 for the reference (**NBR**) and designed molecules (**NBD1-NBD7**).

$$IP = -E_{HOMO} \tag{S1}$$

$$EA = -E_{LUMO} \tag{S2}$$

$$X = \frac{[IP + EA]}{2} \tag{S3}$$

$$\eta = \frac{[IP - EA]}{2} \tag{S4}$$

$$\mu = \frac{E_{HOMO} + E_{LUMO}}{2} \tag{S5}$$

$$\omega = \frac{\mu^2}{2\eta} \tag{S6}$$

$$\sigma = \frac{1}{2\eta} \tag{S7}$$

$$\Delta Nmax = \frac{\mu}{\eta} \tag{S8}$$
